# Photochromic metal–organic frameworks for inkless and erasable printing†
†The synthesis and photochromic property of Mg–NDI and Ca–NDI was previously reported by us^11^ and by Han *et al.*,^12^ respectively.
‡
‡Electronic supplementary information (ESI) available: Contains detailed synthetic procedures, PXRD, FT-IR, TGA, crystallographic data (CIF) and other characterization data. CCDC 1412539. For ESI and crystallographic data in CIF or other electronic format see DOI: 10.1039/c5sc04450b


**DOI:** 10.1039/c5sc04450b

**Published:** 2015-12-21

**Authors:** Bikash Garai, Arijit Mallick, Rahul Banerjee

**Affiliations:** a Physical/Materials Chemistry Division , CSIR-National Chemical Laboratory , Dr. Homi Bhabha Road , Pune-411008 , India . Email: r.banerjee@ncl.res.in; b Academy of Scientific and Innovative Research (AcSIR) , New Delhi , India

## Abstract

Inkless and erasable printing is the key solution towards a more sustainable paper industry, in terms of reducing paper wastages and the associated environmental hazards from waste paper processing. However, only a few cases have been reported in the literature where inkless printing has been tested in some practical systems. In an attempt to address this solution, we used photochromic metal–organic frameworks (MOFs) and tested their capability as inkless and erasable printing media. The printing was performed using sunlight as the light source on MOF-coated papers. The resulting printing had good resolution and stability, and was capable of being read both by the human eye and smart electronic devices; furthermore, the paper could be reused for several cycles without any significant loss in intensity. Interestingly, different coloured printing with a similar efficiency was achieved by varying the structure of the MOF.

## Abstract 2

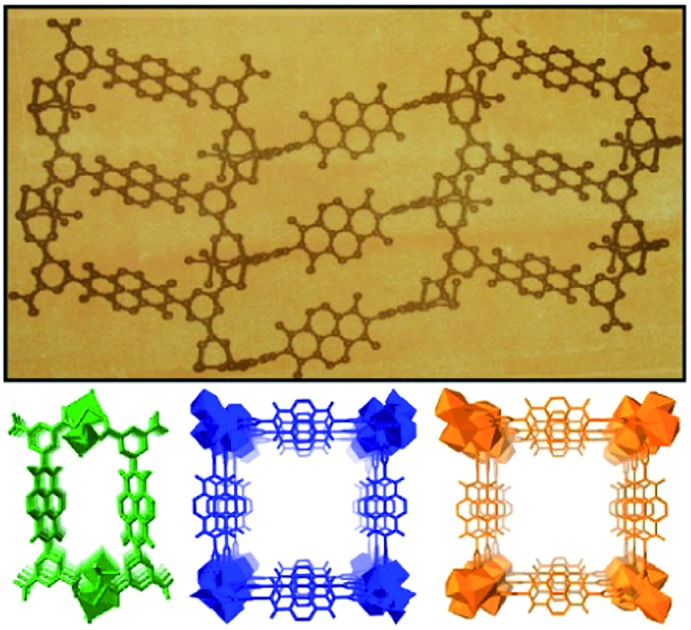
A media for inkless and erasable printing has been developed using photochromic MOFs. Different coloured printing has been achieved by varying the structure of the MOF. The resultant printing has a good resolution and stability, is capable of being read both by human eyes and smart electronic devices and the paper can be reused for several cycles without any significant loss in intensity.

## Introduction

Photochromic materials are capable of changing their colour when exposed to light. Such photochromic materials contain spiropyran, diarylethene, azobenzene or redox active cores in their structures,^1^ which are responsible for the colour change. These materials have been extensively used for making photochromic glasses, lenses and filters because of their interesting reversible colour change property.^2^ Moreover, these materials have also been proposed for applications like erasable and inkless printing, 3D data storage, *etc.*^2^ Inkless and erasable printing is one of the key solutions to address the environmental problems arising because of the ever increasing usage of printed content. Inkless property is necessary to reduce the cost and environment hazards arising from the usage of inks. Reusability of the printing medium is another solution for reducing the paper wastes arising out of printing for temporary purposes. In combination, a potential solution towards sustainability is the use of a print medium that does not require any ink for printing and that could be used over multiple cycles. Although thermal printing is a traditional method for inkless and erasable printing, because of its high energy consumption and sensitivity towards minute heat generation, searches are ongoing for other techniques. The use of a photochromic medium is of potential interest for addressing this problem; however, conventional photochromic materials have short lifetimes and return to their initial colour within a few minutes of excitation. This fast reversibility of these photochromic materials limits their use as print media for inkless and erasable printing where the printed content would vanish in the background. Thus, it is impractical to use these materials as print media for inkless and erasable printing. To avoid such circumstances, the necessary conditions to design practical erasable printing medium are: (i) their ability to retain the photogenerated colour for a prolonged period of time so that the content remains legible/readable; (ii) the reversibility of this colour change so that the same paper can be used for multiple cycles and (iii) the intactness of the colour in the presence of the paper content. Thus, there is a constant search for a suitable photochromic material that could fulfil all the above-mentioned requirements and that could be used for practical applications in inkless and erasable printing media.^3^

Herein, we report three different metal–organic frameworks (MOFs)^4^ constructed from co-ordination between alkaline earth metal ions and ligands containing a photochromic 1,4,5,8-naphthalenediimide (NDI) core^5^ and their application for inkless and erasable printing. NDI has a redox active core and can exhibit reversible photochromism when suitable substituents are attached. In order to avoid the fast decolouration of NDI containing chromophores, we incorporated this NDI core inside the extended structure of MOFs.^6^ We believe that because of the formation of this extended structure and the additional interactions, the photochromic behaviour of the NDI core changes abruptly compared to the discrete NDI units and the system becomes suitable for application as inkless printing media.^3^

## Results and discussions

**Fig. 1:**
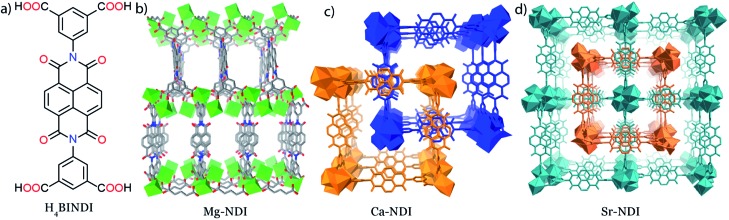
(a) Chemical diagram of H_4_BINDI ligand; crystal structure for (b) Mg–NDI, (c) Ca–NDI and (d) Sr–NDI. Mg–NDI shows a 3D structure, while for the other cases, two nets interpenetrate forming a two-fold interpenetrated structure.

All the MOFs reported in this paper have been synthesized by the solvothermal reactions between the organic BINDI linker (Fig. 1a) and the corresponding metal salts. All these MOFs have a 3D extended structure (Fig. 1b–d), where metal ions are co-ordinated to the four carboxylate groups of the organic ligand. Mg–NDI crystallizes in the *P*2/*c* space group with two different types of co-ordination environment around the Mg(ii) centres. Such co-ordination makes the parallel-orientated NDI moieties become separated by a distance of 7.1 Å. Rectangular-shaped channels [10.9 × 7.1 Å^2^] were generated inside the Mg–NDI structure and the wall of these channels were constructed from NDI moieties (Fig. S3‡). Ca– and Sr–NDI crystallize in the *I*4_1_/*a* space group and differ structurally from Mg–NDI. Both Ca– and Sr–NDI are isostructural, and in the extended framework, two equivalent nets were interlocked *via* π–π stacking between adjacent NDI moieties forming a two-fold interpenetrated structure (Fig. 1c and d). In this case, the NDI moieties of the 2nd net align in a perpendicular orientation compared to the 1st net, during the interpenetration (Fig. S5‡). PXRD patterns of the as-synthesized MOFs indicate the bulk phase purity of the as-synthesized materials (Fig. S6 and S7‡). FT-IR analyses for the MOFs show that additional peaks appear at 2918 and 2846 cm^–1^ in comparison to free ligand; corresponding to the formation of new M–O bonds (Fig. S8‡). The weakly co-ordinating solvents present in the Mg–NDI framework were released around 150 °C temperature, as evidenced from the TGA plots (resulting in a 17% weight loss) of the dried MOF samples and the frameworks eventually decompose at 550 °C (Fig. S12‡).

**Fig. 2:**
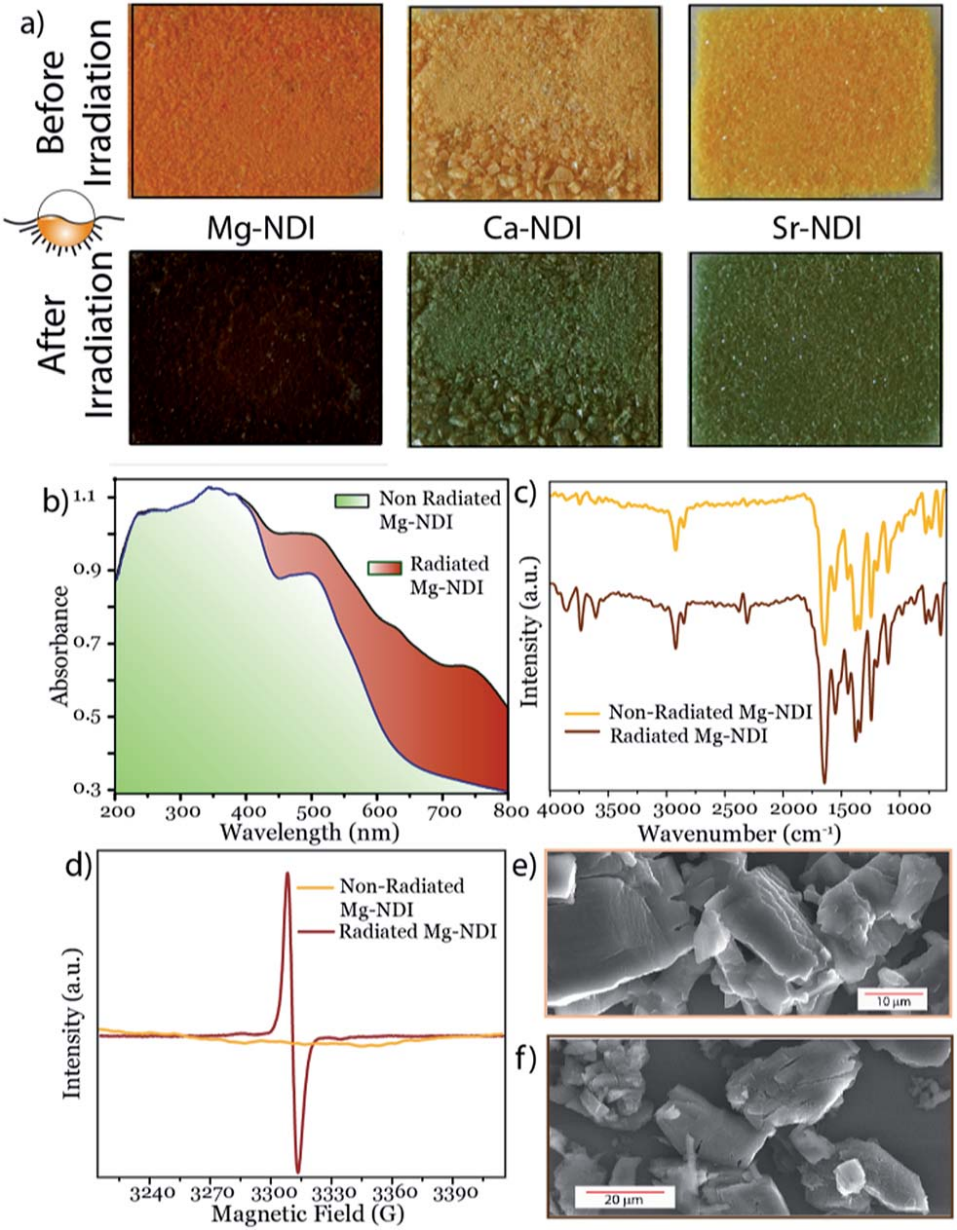
(a) Colour changes of pristine MOF materials under sunlight irradiation, showing their photochromic property; change in (b) UV-vis spectra, (c) IR spectra (d) EPR spectrum, and SEM image (e) before and (f) after sunlight irradiation on Mg–NDI.

As-synthesized Mg–NDI is light yellow in colour while the other MOFs are found to be almost colourless. When these dried MOF crystals were exposed to intense sunlight for 60 s, a drastic colour change occurred for all the MOFs, revealing their photochromic nature (Fig. 2a). The colour of these irradiated materials was found to be dependent on the structure of the MOF. Mg–NDI turned into a brownish black colour after sunlight irradiation, while the isostructural Ca–, and Sr–NDI MOFs turned green after similar treatment. This colour change causes the generation of additional peaks in the UV-vis spectra [630 and 740 nm centred broad peak for Mg–NDI, Fig. 2b; 620 and 605 nm centred peak for Ca– and Sr–NDI, respectively; Fig. S13 and S14‡]. The observed PXRD patterns (Fig. S6 and S7‡) indicate that these MOFs retain their initial structure even after the photochromic transformation and corresponding quenching. FT-IR spectra of all the MOFs were also found to be identical for the cases of non-radiated, radiated and quenched materials, again suggesting the retention of functional groups and the bonding during this photochromic change (Fig. 2c). Apart from the internal structure, the external morphology of Mg–NDI was also retained after sunlight irradiation, as evidenced from the SEM images (Fig. 2e and f).

It is well documented in the literature that because of the n-type character, under suitable conditions the NDI moiety undergoes one electron transfer and is converted into an NDI radical species (NDI˙).^7^ This NDI˙ can be generated from neutral NDI *via* various methods, like chemical, photochemical and electrochemical treatment.^8^ NDI˙ bears a characteristic EPR signal in the *g* = 2.002–2.004 region, originating from the unpaired electron.^9^ This unpaired electron from NDI˙ gets quenched readily when it comes into contact with paramagnetic species like oxygen gas.^7^ The use of alkaline earth metal ions as nodes for the construction of Mg–, Ca– and Sr–NDI keeps the unpaired electron of the radical species unaffected due to the absence of a partially filled d-orbital. EPR studies revealed that as-synthesized MOFs are silent to the applied magnetic field while a sharp singlet peak appears in the case of sunlight-radiated MOFs with *g* = 2.003 (Fig. 2d, S16 and S17‡). This signal accounts for the formation of NDI˙ radical species upon sunlight radiation, which vanishes when the materials are quenched and returned to their original colour. It is noteworthy that after sunlight irradiation, the BINDI ligand also shows a similar signal [*g* = 2.004, Fig. S18‡] in the EPR spectrum; indicating the generation of NDI˙ species in its structural backbone. However, this radical generation requires a prolonged irradiation time [10 min] followed by quick loss of photogenerated colour [reverts to original colour in <2 h, Fig. S20‡], indicating a shorter lifetime [compared to 12 h for Mg–NDI] for the generated radical species. It was found that the colour of the radiated material has a poor contrast compared to the non-radiated one, thus making BINDI non-suitable for inkless and erasable printing applications. This short lifetime of the BINDI radical species is a result of the short π–π stacked NDI cores, which are located at a separating distance of 2.6 Å, as evidenced from its crystal structure (Fig. S4‡). Stacking between the adjacent NDI cores facilitates the quenching of NDI˙ through the transfer of electrons to the neighbouring moieties. As a result, the photogenerated colour becomes transient and quickly reverts to its initial colour. Interestingly, these NDI cores are separated by a distance of ∼7.1 Å for Mg–NDI, which eliminates the chance of π–π stacking among those moieties and brings stability to the NDI˙ radical species (Fig. S3‡). It has been evidenced in the literature that the mobility of the radical electron from the NDI core is enhanced in the solid state when they are perfectly stacked over each other, but, when the stack is removed by solubilising in proper solvent, the transport of the electron is hampered. Thus, localization of the radical has been achieved by separating the NDI cores in solution.8a,^10^ In the case of isostructural Ca– and Sr–NDI MOFs, the π–π stacking distance between the adjacent NDI moieties is 2.4 Å. However their orientation is orthogonal to each other (Fig. S5‡). Furthermore, because of this orthogonal orientation, radicals cannot be quenched *via* a transport mechanism as occurs with the bare BINDI ligand. Thus the photogenerated NDI˙ within the MOF backbone attains stability. The nature of the photogenerated radical [singlet peaks centred at *g* = 2.003] was found to be the same for all the MOFs (Fig. S19‡) and for the bare BINDI ligand.

**Fig. 3:**
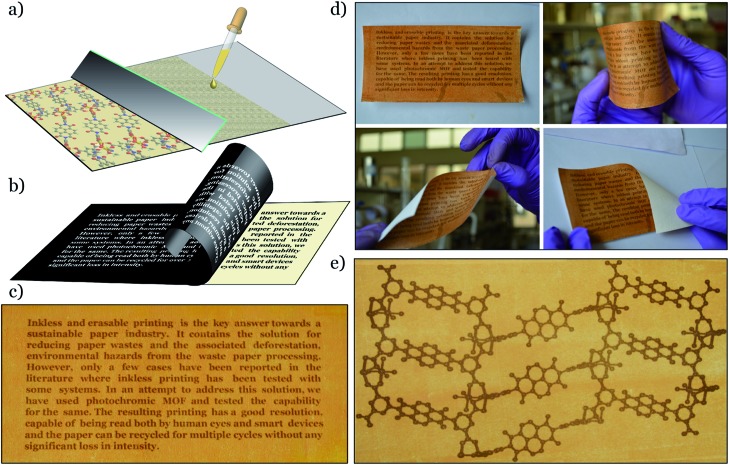
(a) Schematic representation for the preparation of Mg–NDI coated paper; (b) scheme for printing on the coated paper with stencil and sunlight; (c) test for resolution of the printed content by the printing of letters on a 11.5 × 5.4 cm^2^ paper; (d) test for mechanical deformation with Mg–NDI coated paper and (e) image showing ball and stick model of Mg–NDI structure on the Mg–NDI coated paper having a dimension of 14.9 × 8.1 cm^2^.

Noting this interesting photochromic property of these NDI-based MOFs, we planned to use them as inkless and erasable printing media. The MOF-coated paper was prepared by drop-casting an ethanol suspension of finely powdered Mg–NDI on a cellulose filter paper, followed by surface smoothening with a glass slide (Fig. 3a). The paper was then dried under vacuum, where the coating was adhered to the paper, without losing the flexible nature of the resulting coated paper (Fig. 3d). The printing of the contents on this coated paper was performed by controlling the incidence of sunlight through a stencil. The stencil was prepared by printing an inverted object of the desired content on a transparent polyurethane sheet (section S14 in ESI). The printing surface of the coated paper was then covered with the stencil and the assembly was kept in intense sunlight (having a flux of 100 mW cm^–2^) for less than 60 s. After this exposure, the stencil was removed from the top of the coated paper to obtain the content printed in a brownish black colour on a pale yellow background of Mg–NDI (Fig. 3b). Large-scale text printing was tested with a stencil of an 11.9 × 5.4 cm^2^ sized print obtained with a similarly designed stencil. No overlap among the 610 characters occupied in 10 lines was observed and each of the characters was clearly distinguishable from its next neighbour (Fig. 3c). The colour contrast between the foreground and background was found be good enough for visual reading of the content. This visual legibility was again confirmed from an outline sketch with a dimension of 14.9 × 8.1 cm^2^. The objects present in the drawing were well-defined in respect to their constituent lines and the printed content could be easily visualized (Fig. 3e). Similar printing with Ca– and Sr–NDI coated paper gave excellent legibility, where the resulting content was printed in a dark green colour.

**Fig. 4:**
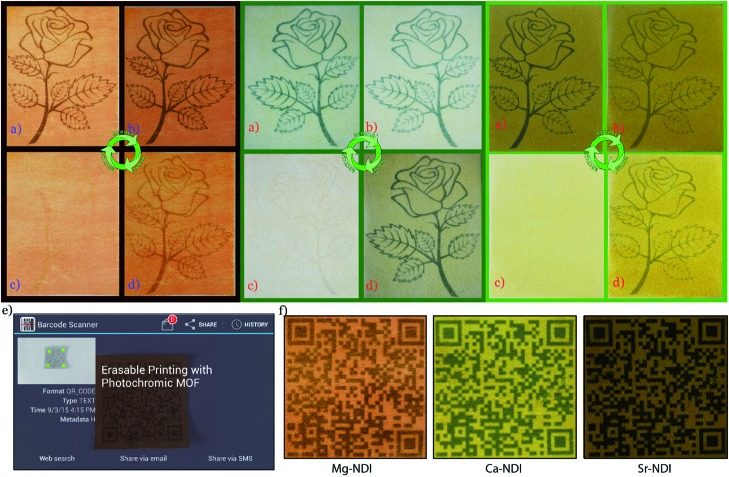
(a) Photograph of content printed on Mg–NDI coated paper; (b) content after 12 h of printing; (c) self-erased paper after keeping in the dark for 12 h; (d) photograph of the paper after printing for the 4th round; (e) detection of a QR code printed on the Mg–NDI coated paper with a smartphone; (f) QR code printed on Mg–NDI, Ca–NDI and Sr–NDI coated paper showing different coloured printing.

The printed content was found to disappear into the background after 24 h for the cases of Mg–, Ca– and Sr–NDI coated papers, converting them into a blank paper which could be used for the next round of printing and, as shown in Fig. 4d, the intensity of the 4th round printed content remained comparable to the 1st round, though some loss in contrast with the background could be observed because of the repeated cycles. As the printed content was kept in ambient atmosphere, aerial oxygen diffused through the excited material, converting it back to the initial state. The time required for complete quenching of the excited material along with complete reversal to the initial colour was found to be 24 h. Thus, the printed content remained legible for a long period of time (Fig. 4c), at least enough for temporary uses. It is noteworthy that the erasing of this printed paper can be accelerated for reuse during this 24 h period by flushing oxygen gas onto the printed paper. Thus, reversibility and recyclability of the printing media were successfully established.

Apart from the naked eye legibility of the printed content, the resulting printing was found to have enough resolution to be recognized by smart devices. 1D and 2D barcodes were printed on the Mg–, Ca– and Sr–NDI coated papers using an identical stencil, to confirm the ability to be decoded by smart devices. A version-5 QR code (containing 37 rows and 37 columns) with a dimension of 4.7 × 4.7 cm^2^ was prepared that contained 39 characters (Fig. 4f). The embedded code ‘Erasable Printing with Photochromic MOF’ was readily decoded with any reader software installed on smart electronic devices, as shown in Fig. 4e. A similar quick response was also found for the case of 1D barcodes (Fig. S23‡), proving the excellent machine legible nature of the printed content on the Mg–NDI coated paper. A UV-vis study of the printed and erased papers over multiple cycles (Fig. S15‡) showed that the colour intensity of Mg–NDI in both the coloured and colourless form held steady for 04 cycles.

## Conclusions

In conclusion, we demonstrated a novel approach to develop an inkless and erasable printing medium using photochromic MOFs. Precise impression of the desired content on the print medium was achieved by controlling the incidence of sunlight on the print medium with a stencil and without use of any ink. Furthermore, the resulting print was well-recognized by smart electronic devices as well. The printed content was self-erased after 24 h, without using any other external stimuli like heat or UV light. The self-erasing nature makes the system suitable for performing several printing–erasing cycles with the same paper, making the printing process cost-effective and environmentally friendly. In addition, we could tune the colour of printing by selecting different MOFs having different structures. Development of new materials capable of showing multicolour-photochromic behaviour for application in colour printing is underway in our laboratory.

## Supplementary Material

Supplementary information

Crystal structure data
