# Supplementary material for: Photochromic metal–organic frameworks for inkless and erasable printing
Source: Chem Sci. 2015 Dec 21;7(3):2195–200. doi: 10.1039/c5sc04450b (PMC6063288; doi:10.1039/c5sc04450b)
Supplement: Supplementary file 1 [file SC-007-C5SC04450B-s001.pdf]

Electronic Supplementary Material (ESI) for Chemical Science.
This journal is © The Royal Society of Chemistry 2015

Electronic Supporting Information

Photochromic Metal-Organic Frameworks for Inkless and Erasable Printing

Bikash Garai,[a,b] Arijit Mallick,[a,b] and Rahul Banerjee[a,b]*

[a]*Physical/Materials Chemistry Division, CSIR- National Chemical Laboratory, Dr. Homi Bhabha Road, Pune 411008, India.*

[b]*Academy of Scientific and Innovative Research (AcSIR), New Delhi, India*

Email: r.banerjee@ncl.res.in; Fax: + 91-20-25902636, Tel: + 91-20-25902535

Table of Contents

Section	Subject	Page
S1	Materials and methods	S3
S2	Synthesis of BINDI ligand and metal organic frameworks	S4
S3	Single crystal XRD and crystal structure of **Sr-NDI**	S5
S4	PXRD patterns of the materials	S12
S5	FT-IR spectroscopy of BINDI linker and derived MOFs	S14
S6	TG analyses of the materials	S18
S7	UV-vis spectroscopic measurements	S19
S8	Electron Paramagnetic Resonance (EPR) studies	S22
S9	Photochromic property of BINDI	S26
S10	SEM micrograph of Mg-NDI coated paper	S27
S11	Resolution test of printing with Mg-NDI coated paper	S28
S12	Detection of 1D Barcode	S29
S13	Stability of the Mg-NDI coated paper towards mechanical deformation	S30
S14	Preparation of stencils	S31
S15	References	S32

S1: Materials and methods

All reagents were commercially available and used as received without any further purification. Single Crystal X-Ray Diffraction data were collected on a Super Nova Dual source X-ray Diffractometer system (Agilent Technologies) equipped with a CCD area detector. Powder X-ray diffraction (PXRD) patterns were recorded on a Rigaku Smartlab diffractometer for Cu K_{α} radiation (λ = 1.5406 Å), with a scan speed of 2° min^{-1} and a step size of 0.02° in 2θ. Fourier transform infrared (FT-IR) spectra were recorded on a Bruker Optics ALPHA-E spectrometer with a universal Zn-Se ATR (attenuated total reflection) accessory in the 600-4000 cm^{-1} wavenumber region. Thermo-gravimetric analyses (TGA) were carried out on a SDT Q600 TG-DTA analyzer under N_2 atmosphere at a heating rate of 10 ºC min^{-1} within the temperature range of 30-900 °C. Solid state UV-vis absorbance studies were carried out with a Agilent make UV-vis-NIR spectrophotometer. EPR measurements were performed with a Bruker instrument under ambient atmospheric condition.

S2: Synthesis of BINDI ligand and metal organic framework

N,N'-bis(5-isophthalic acid)naphthalenediimide (H_4BINDI) was synthesized following a previously reported procedure in the literature[1]. 1,4,5,8-naphthalene-tetracarboxylic acid dianhydride (6.70 g, 25.0 mmol) was taken into a 250 mL round bottomed flask and suspended in 125 mL acetic acid. The mixture was stirred for 10 min. To this solution, 5-aminoisophthalic acid (9.05 g, 50.0 mmol) was added and the resulting suspension was allowed to reflux for 12 h. The reaction was cooled down to room temperature and 100 mL of water was added to precipitate the product. The resultant product was collected by filtration and washed with ethanol. The obtained solid was dried in vacuum to yield 12.0 g of off-white solid (isolated yield = 12.0 g, 77%). The compound was recrystallized from DMF as an off-yellow materials (isolated yield = 10.5 g, 67%).

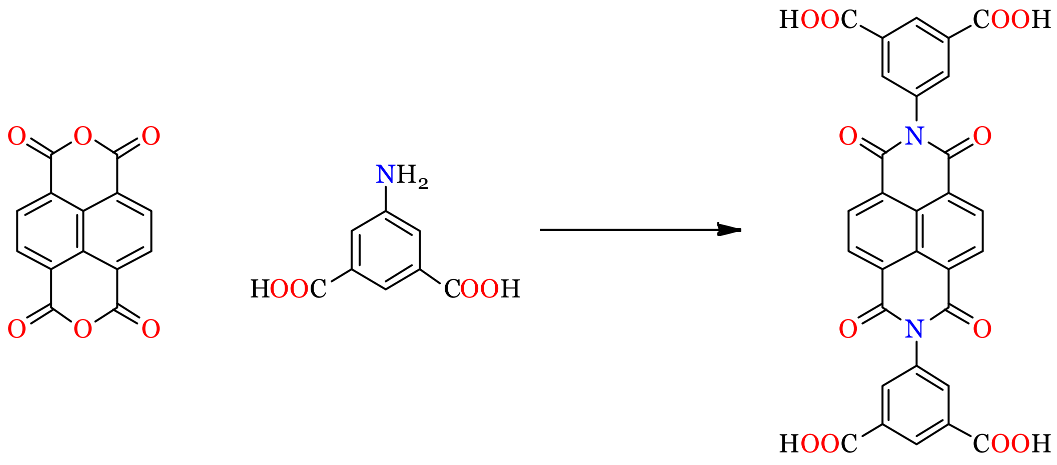

Figure S1: Scheme for synthesis of H_4BINDI from the starting materials.

Synthesis of Sr-NDI: The needle shaped crystals of Sr-NDI MOF was synthesized by reacting BINDI (21 mg, 0.035 mmol) with $Sr(NO_3)_2$ (24 mg, 0.093 mmol) in 4 mL DMF and 0.2 mL HCl (3 N) at 90 ˚C for 24 h. Needle shaped colourless crystals were collected from the reaction vial and washed with dry DMF and preserved for further applications and characterizations.

S3: Single crystal XRD and crystal structure of Sr-NDI

As synthesized crystal of Sr-NDI was placed inside a glass capillary (Hampton research) and then mounted in the diffractometer. The data collection was done at 200 K. The crystals were mounted on a Super Nova Dual source X-ray Diffractometer system (Agilent Technologies) equipped with a CCD area detector and operated at 250 W power (50 kV, 0.8 mA) to generate Mo Kα radiation (λ = 0.71073 Å) and Cu Kα radiation (λ = 1.54178 Å) at 298(2) K. Initial scans of each specimen were performed to obtain preliminary unit cell parameters and to assess the mosaicity (breadth of spots between frames) of the crystal to select the required frame width for data collection. $CrysAlis^{Pro}$ program software was used suite to carry out overlapping φ and ω scans at detector (2θ) settings (2θ = 28). Following data collection, reflections were sampled from all regions of the Ewald sphere to re-determine unit cell parameters for data integration. In no data collection was evidence for crystal decay encountered. Following exhaustive review of collected frames the resolution of the data set was judged. Data were integrated using $CrysAlis^{Pro}$ software with a narrow frame algorithm. Data were subsequently corrected for absorption by the program SCALE3 ABSPACK[2] scaling algorithm.

These structures were solved by direct method and refined using the SHELXTL 97[3] software suite. Atoms were located from iterative examination of difference F-maps following least squares refinements of the earlier models. Final model was refined anisotropically (if the number of data permitted) until full convergence was achieved. Hydrogen atoms were placed in calculated positions (C-H = 0.93 Å) and included as riding atoms with isotropic displacement parameters 1.2-1.5 times Ueq of the attached C atoms. In some cases modeling of electron density within the voids of the frameworks did not lead to identification of recognizable solvent molecules in these structures, probably due to the highly disordered contents of the

large pores in the frameworks. Highly porous crystals that contain solvent-filled pores often yield raw data where observed strong (high intensity) scattering becomes limited to ~1.0 Å at best, with higher resolution data present at low intensity. A common strategy for improving X-ray data, increasing the exposure time of the crystal to X-rays, did not improve the quality of the high angle data in this case, as the intensity from low angle data saturated the detector and minimal improvement in the high angle data was achieved. Additionally, diffused scattering from the highly disordered solvent within the void spaces of the framework and from the capillary to mount the crystal contributes to the background and the 'washing out' of the weaker data. Electron density within void spaces has not been assigned to any guest entity but has been modeled as isolated oxygen and/or carbon atoms. The foremost errors in all the models are thought to lie in the assignment of guest electron density. The structure was examined using the *ADSYM* subroutine of PLATON[4] to assure that no additional symmetry could be applied to the models. The ellipsoids in ORTEP diagrams are displayed at the 50% probability level unless noted otherwise.

It is noteworthy that despite of our several attempts to model the disorder the solvent molecules present inside the MOF pores, we couldn't able to completely assign them as chemical entities. This has lead to the generation of some A and B level errors in the IUCr Checkcif report. This modeling has also caused some checks for C-H bonds which have appeared as A level error and couldn't be removed by refinement. This model has also developed a minute shift in some of the peaks of simulated PXRD pattern; although the experimental PXRD pattern holds good all the cases, proving our hypothesis. Despite of this weak modeling of the non co-ordinated solvent molecules, the provided structure is good enough to describe the structure of the framework.

CCDC 1412539 contains the crystallographic data for Sr-NDI MOF.

Table S1: Crystal data and structure refinement for Sr-NDI

Identification code	Sr-NDI
Empirical formula	C44 H44 N4 O23 Sr2
Formula weight	1172.07
Temperature/K	200.01(2)
Crystal system	tetragonal
Space group	*I* 41/*a*
a/Å	28.4784(7)
b/Å	28.4784(7)
c/Å	13.6334(4)
α/°	90.00
β/°	90.00
γ/°	90.00
Volume/Å^3	11056.9(5)
Z	8
ρ_{calc}mg/mm^3	1.408
m/mm^{-1}	0.109
F(000)	4768
Crystal size/mm^3	0.6 × 0.2 × 0.2
Theta range for data collection	3.16 to 29.08°
Index ranges	$-30 \leq h \leq 38$, $-38 \leq k \leq 38$, $-17 \leq l \leq 18$
Reflections collected	11940
Independent reflections	7392[R(int) = 0.0447]
Data/restraints/parameters	7392/0/367
Goodness-of-fit on F^2	0.930
Final R indexes [I>2σ (I)]	R_1 = 0.1393, wR_2 = 0.3624
Final R indexes [all data]	R_1 = 0.1810, wR_2 = 0.3919
Largest diff. peak/hole / e Å^{-3}	2.08/-1.82

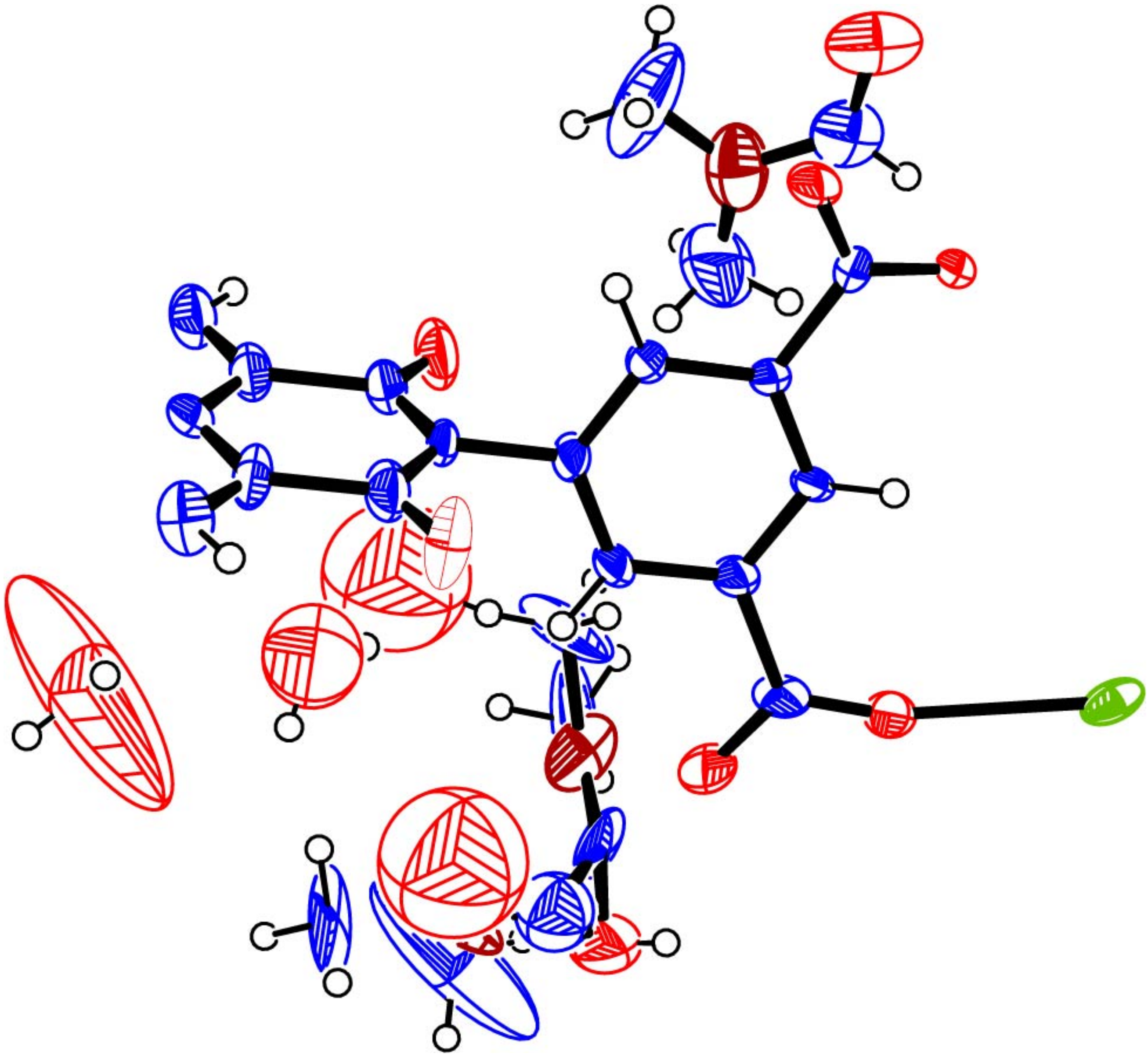

Figure S2: ORTEP drawing of Sr-NDI. Thermal ellipsoids set to 50% probability level.

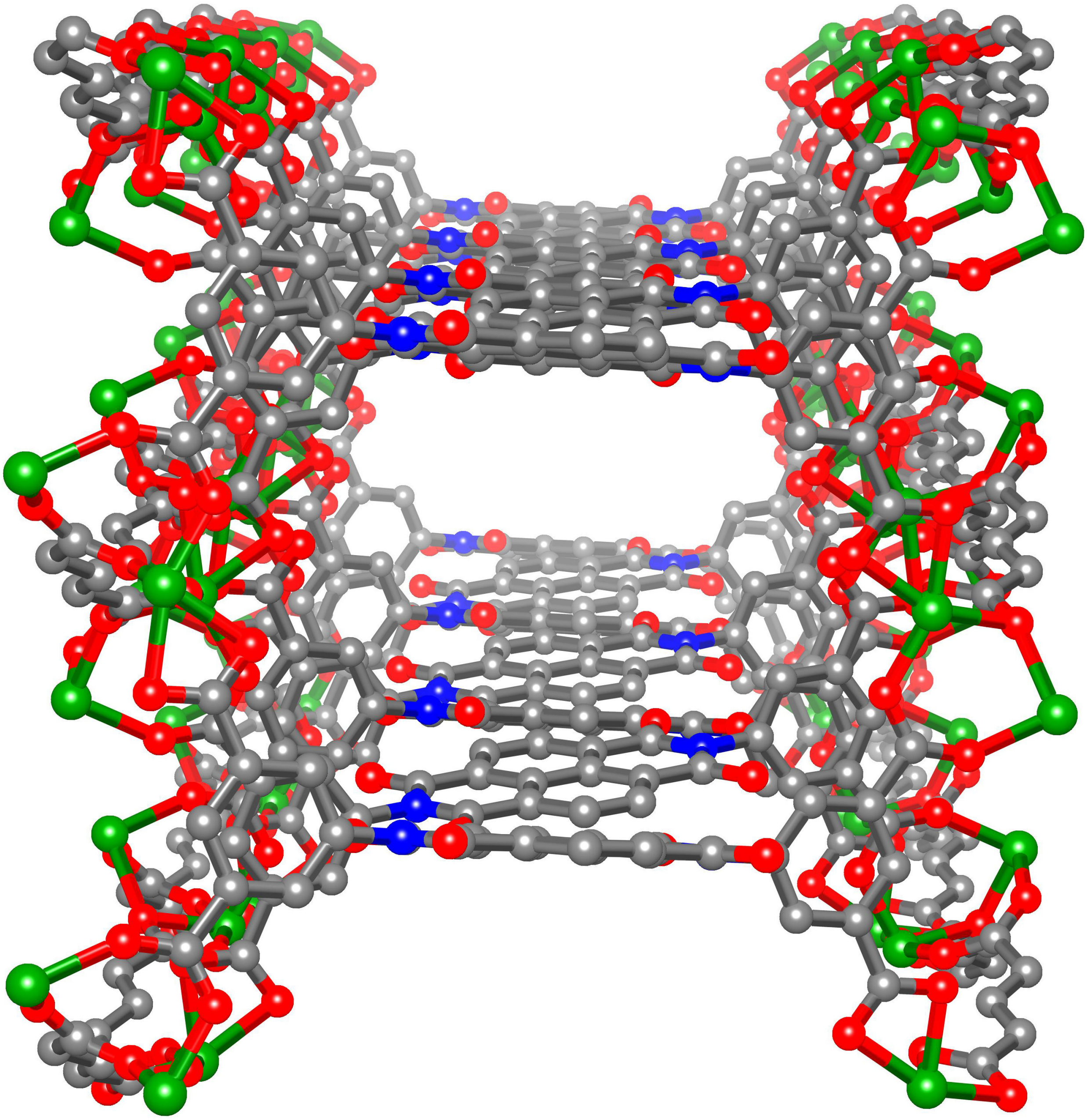

Figure S3: Construction of pore walls of Mg-NDI from naphthalenediimide moieties showing their parallel stacking (distance between two parallel oriented NDI moieties is 7.1 Å).

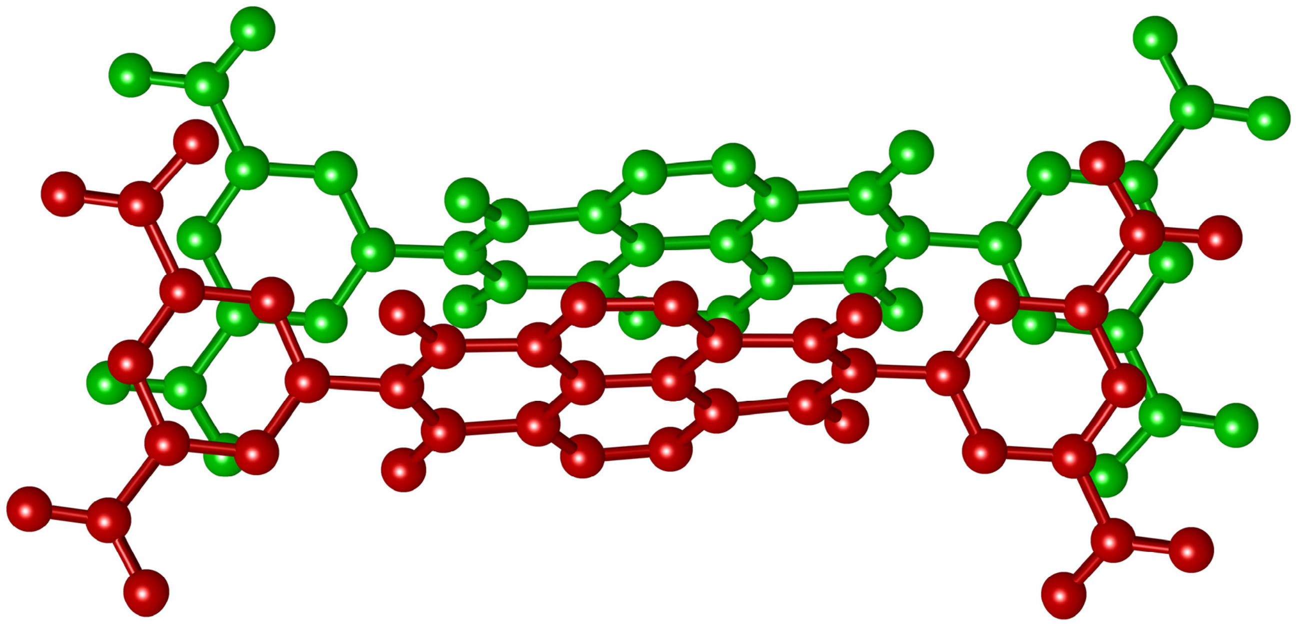

Figure S4: Parallel stacking of NDI moieties in BINDI ligand (distance between two parallel oriented NDI moieties is 2.6 Å).

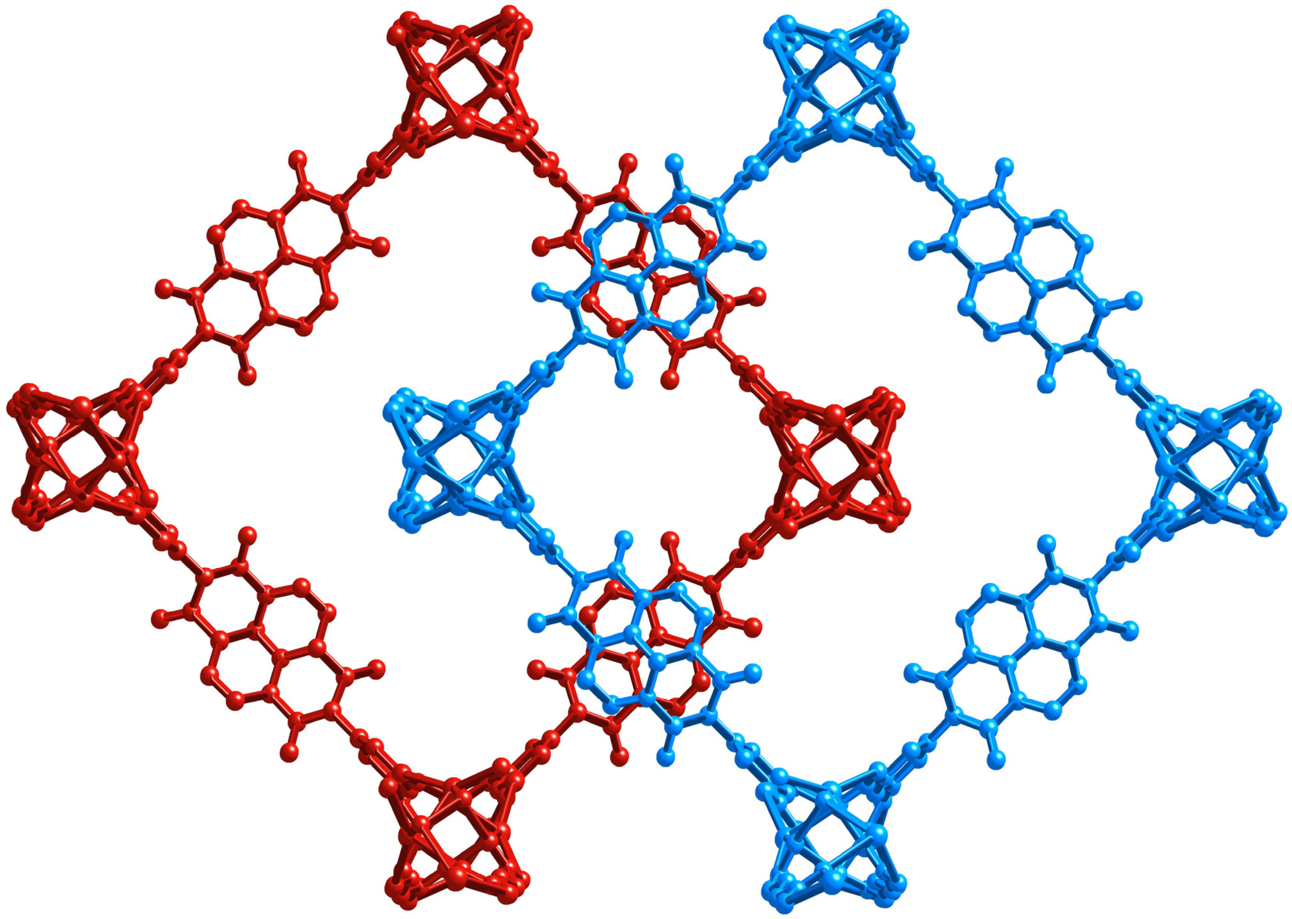

Figure S5: Crystal Structure of Sr-NDI showing orthogonal orientation of NDI moieties from inter-penetrated form (distance between two orthogonally oriented NDI moieties is 2.4 Å).

S4: PXRD patterns of the materials

As synthesized crystals of the MOF materials were taken out from the mother solution and washed with dry DMF and pure ethanol and then dried in vacuum. The dried materials were then placed in the quartz PXRD plates and kept in the powder diffractometer to record their XRD pattern as a tool of checking the bulk phase purity of the MOFs.

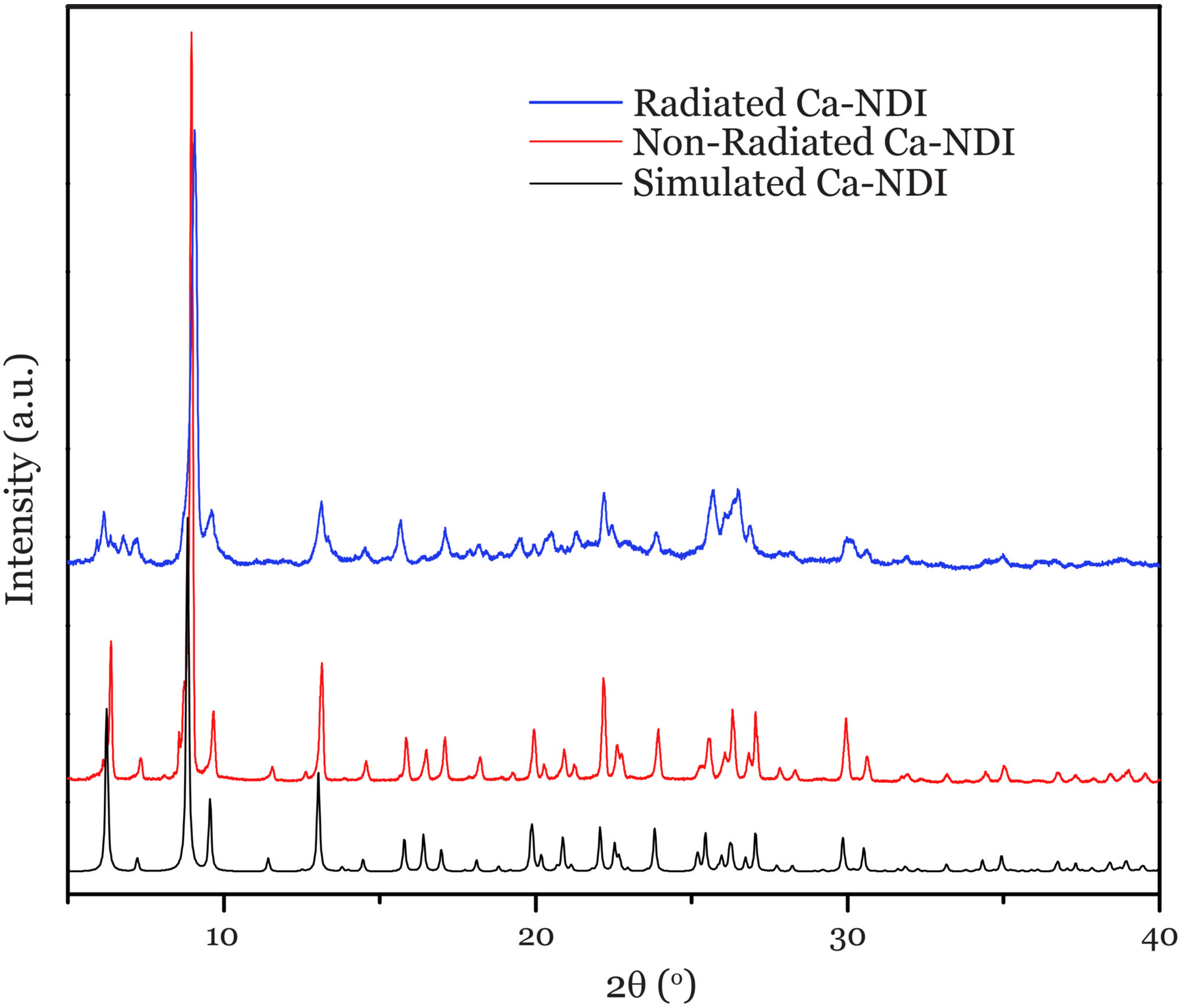

Figure S6: PXRD pattern of Ca-NDI from As-synthesized state (Red) and Radiated State (Blue). Change in background noise has been observed for the case of radiated samples, however the major peaks remained intact in all the cases.

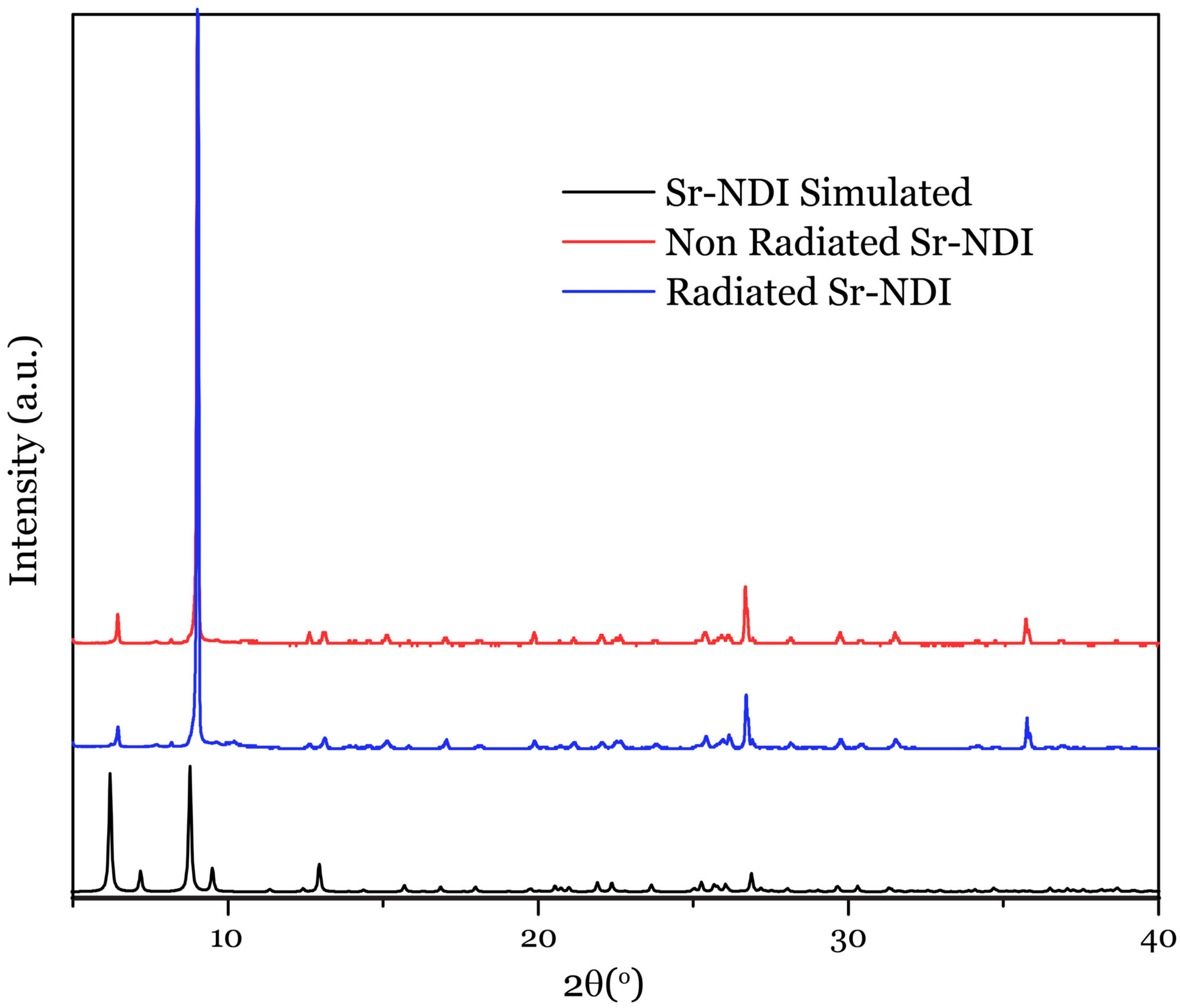

Figure S7: PXRD pattern of Sr-NDI from As-synthesized state (Red) and Radiated State (Blue).

S5: FT-IR spectroscopy of BINDI linker and derived MOFs

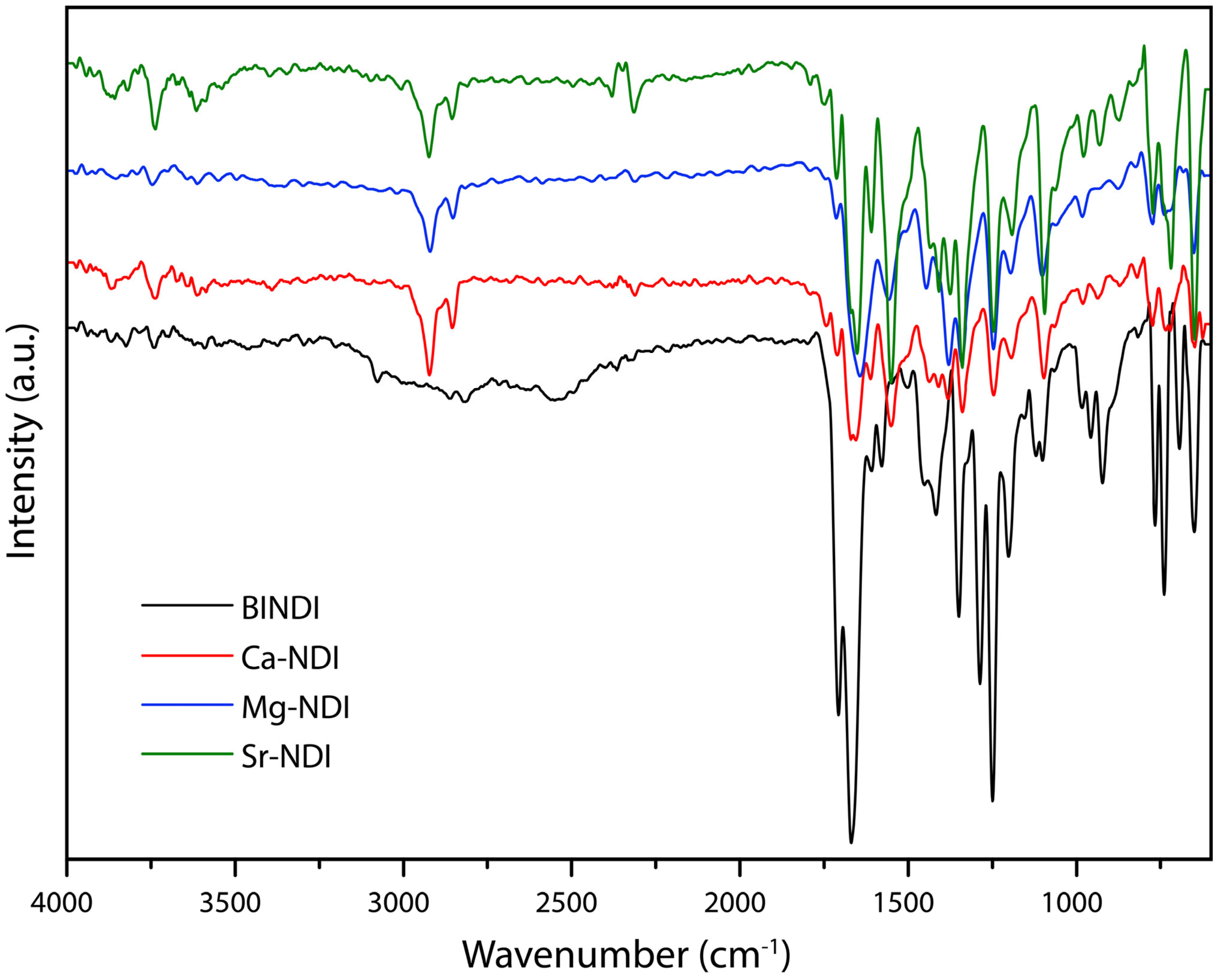

Figure S8: FT-IR spectra of BINDI linker and the derived MOFs.

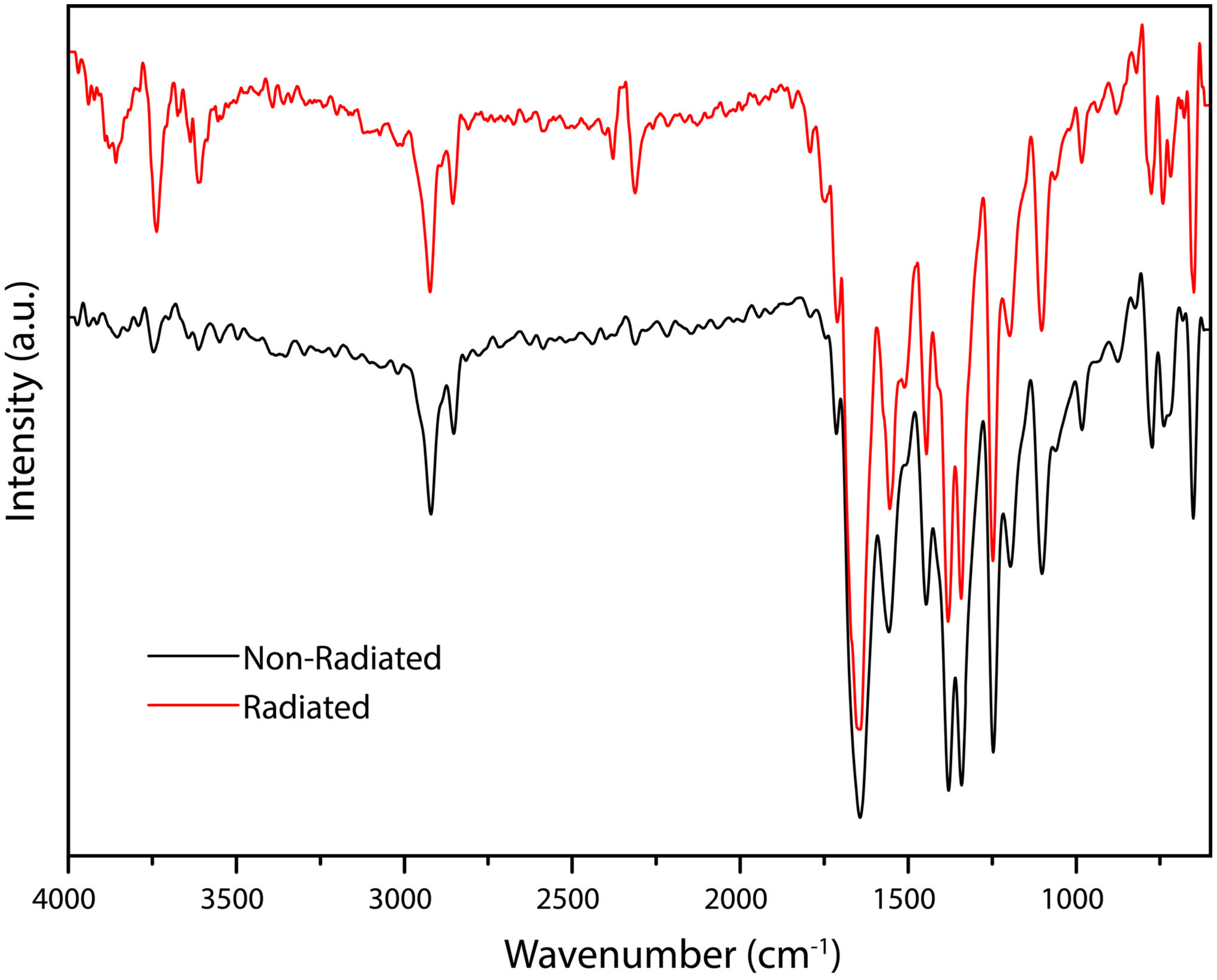

Figure S9: FT-IR spectra for Mg-NDI recorded before and after sunlight irradiation.

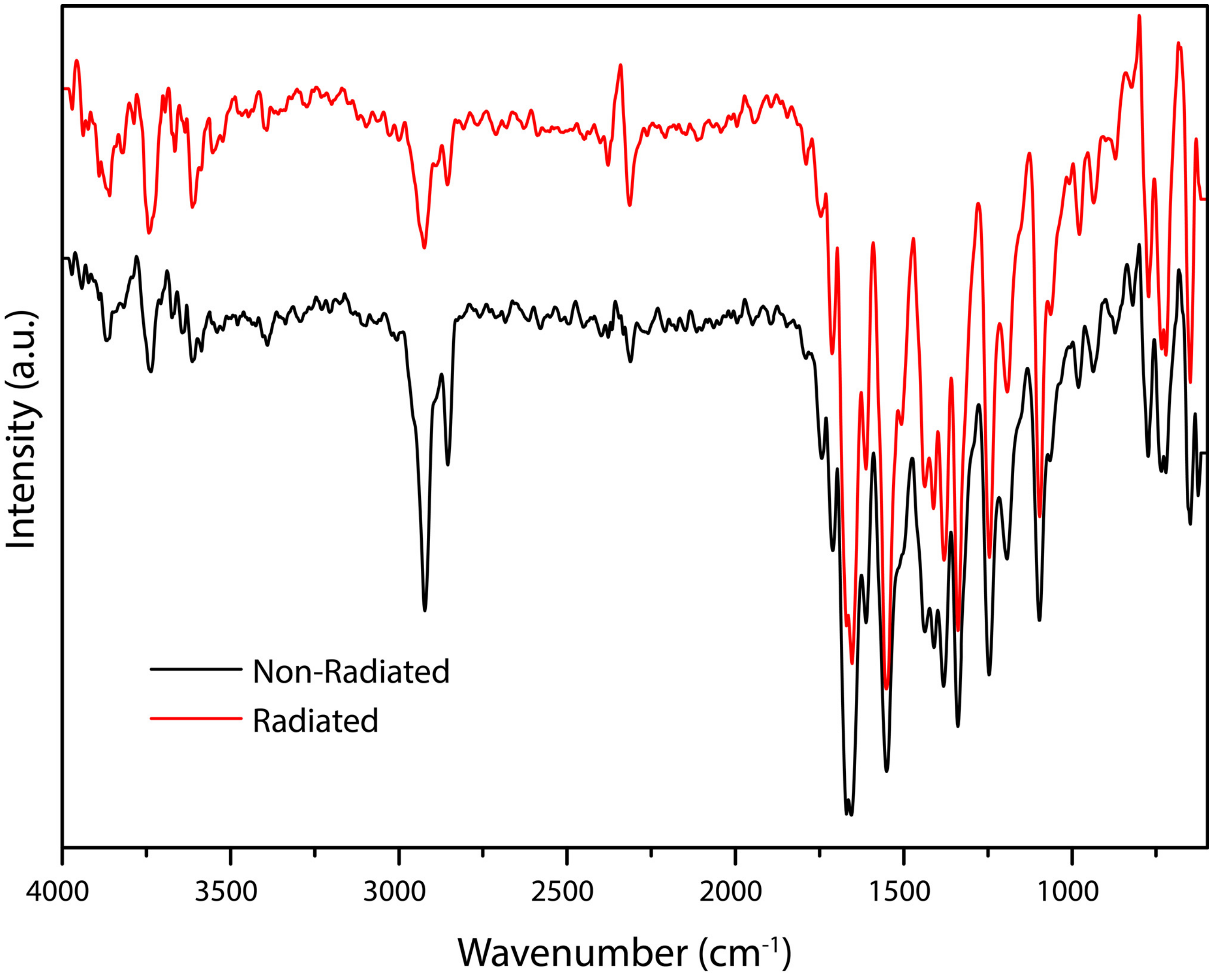

Figure S10: FT-IR spectra for Ca-NDI recorded before and after sunlight irradiation.

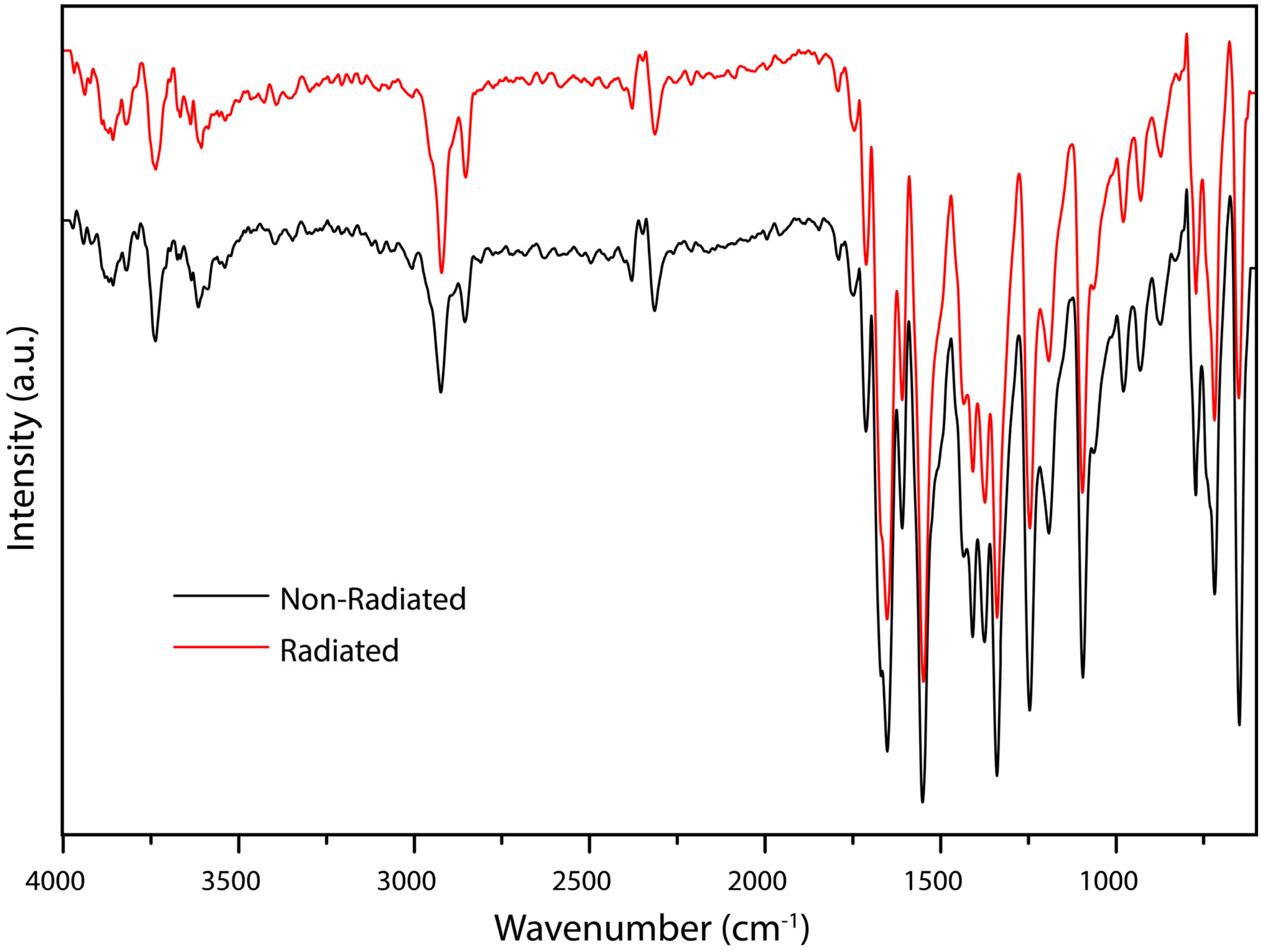

Figure S11: FT-IR spectra of Sr-NDI recorded before and after sunlight irradiation.

S6: TG analyses of the materials

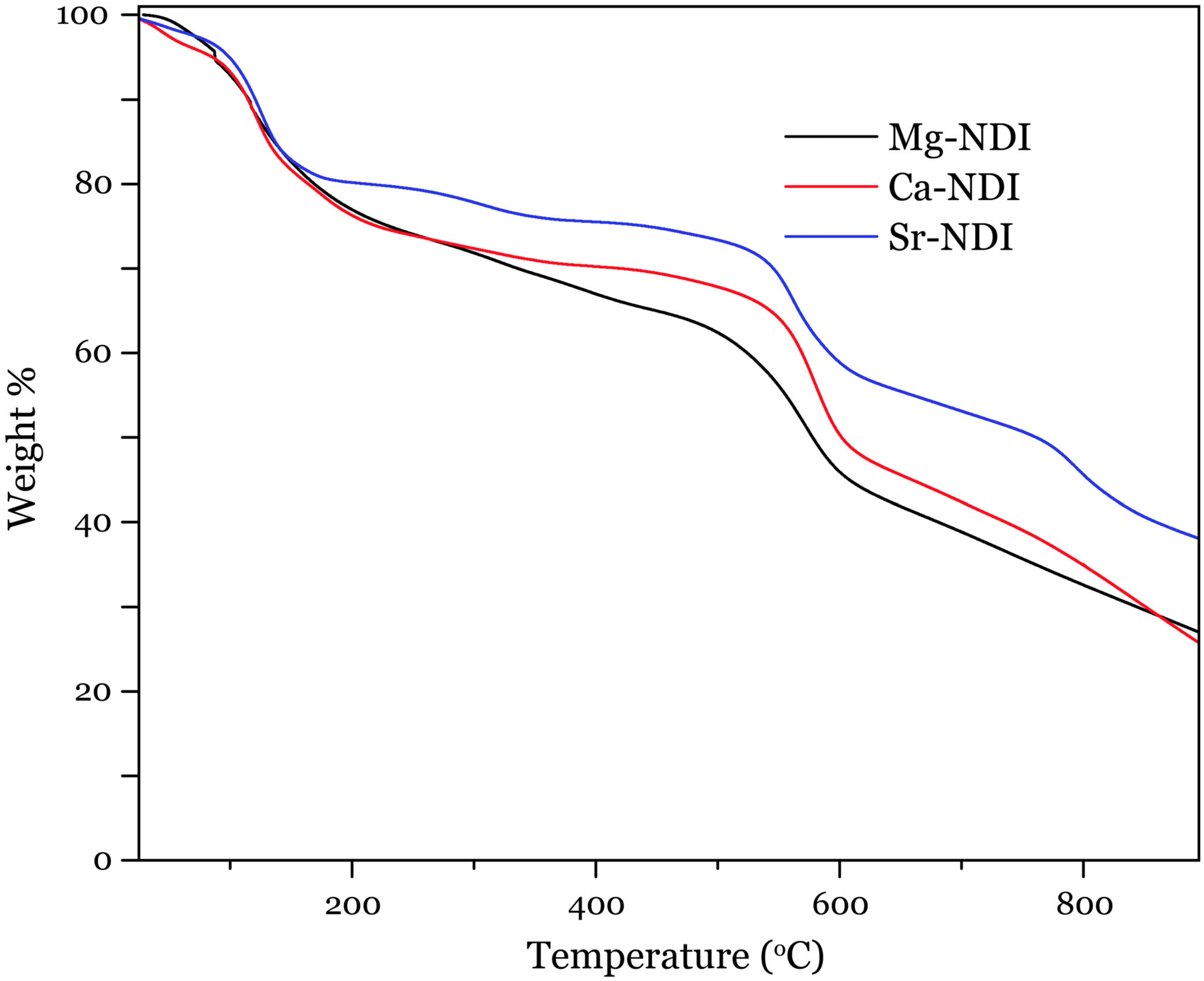

Figure S12: TGA plots of the MOFs showing their thermal stability.

S7: UV-vis spectroscopic measurements

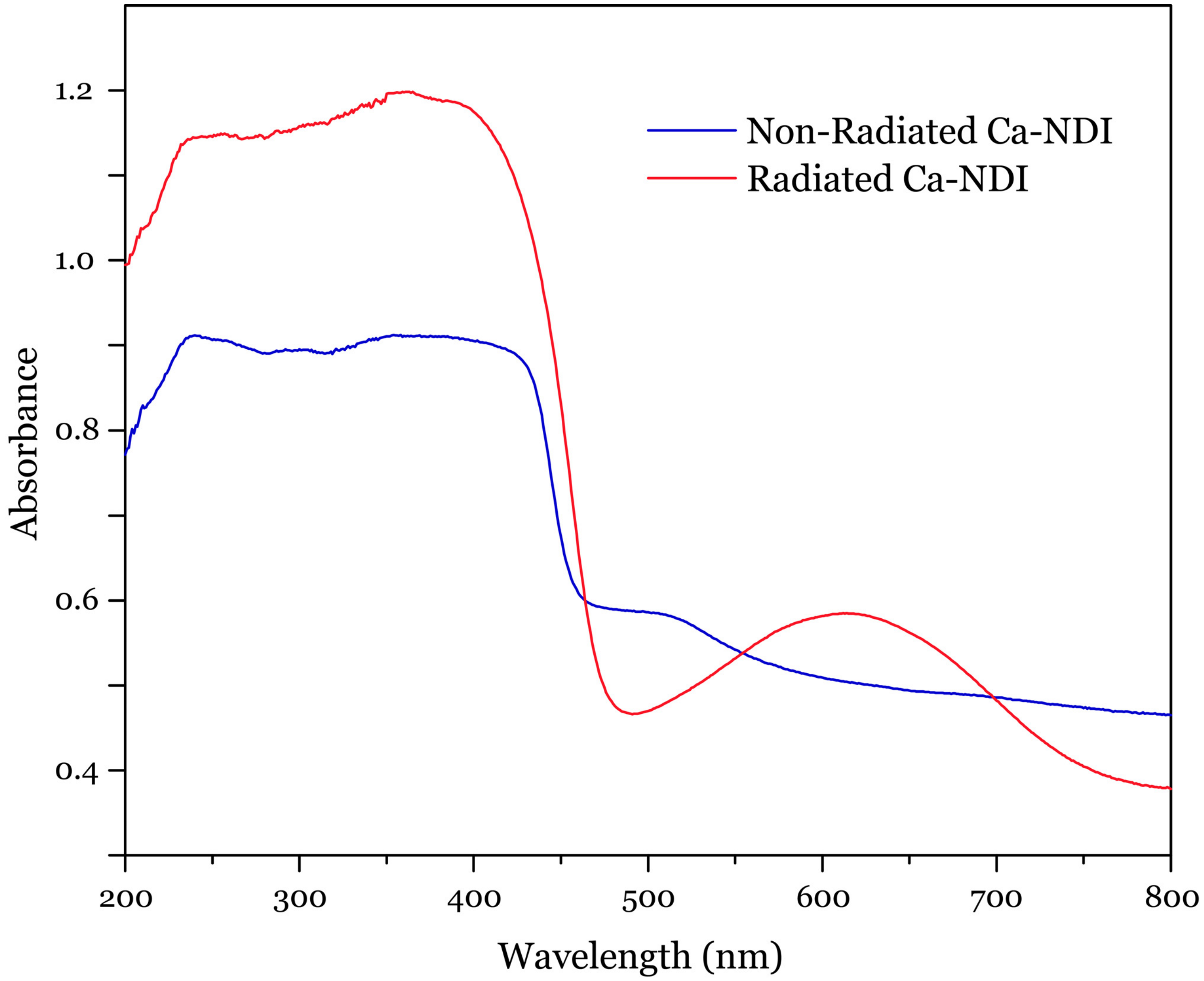

Figure S13: UV-vis spectra for Non-radiated and Radiated Ca-NDI showing generation of new peak centered at 620 nm because of sunlight irradiation.

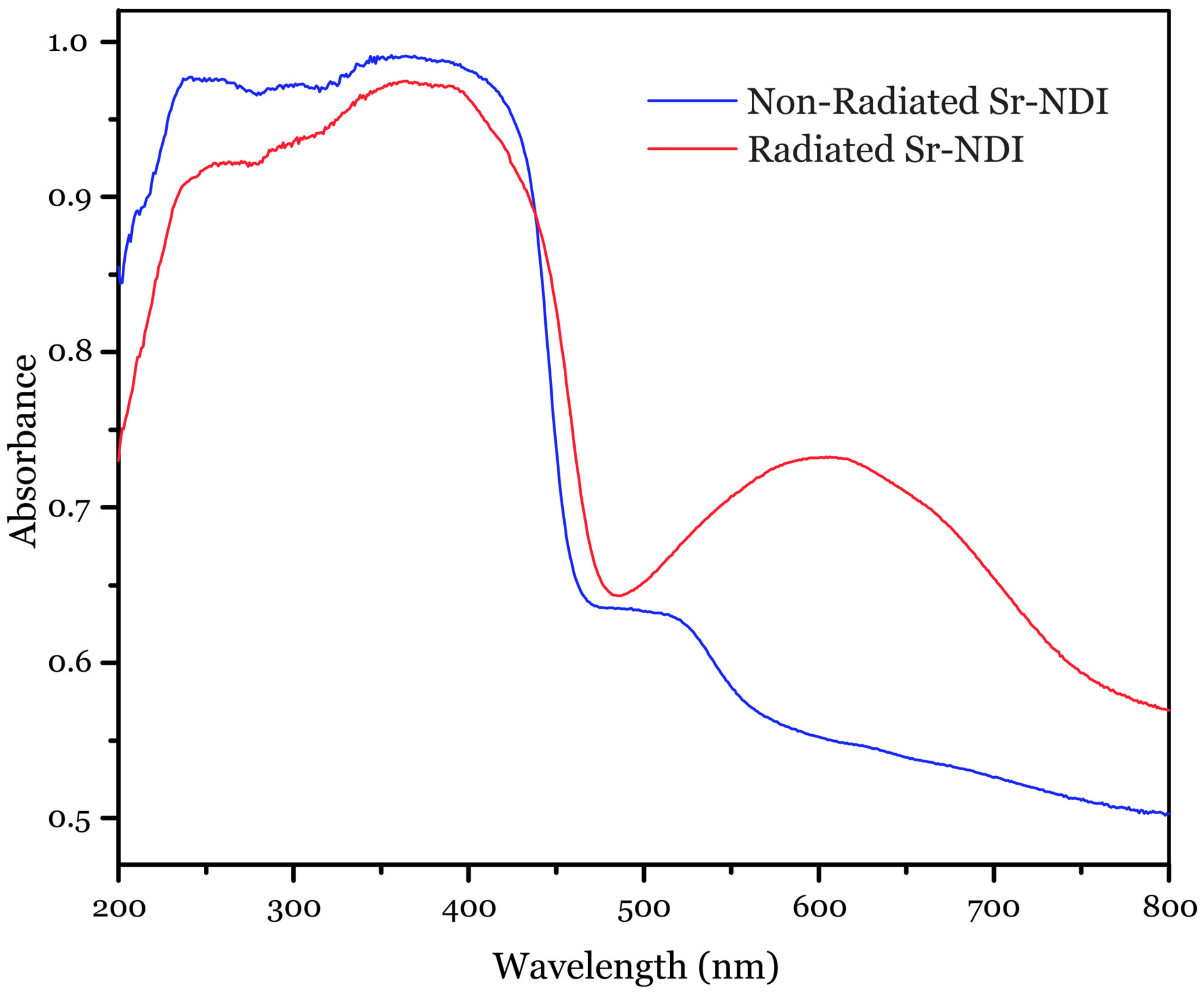

Figure S14: UV-vis spectra of Non-Radiated and Radiated Sr-NDI showing generation of new peak centered at 605 nm because of sunlight irradiation.

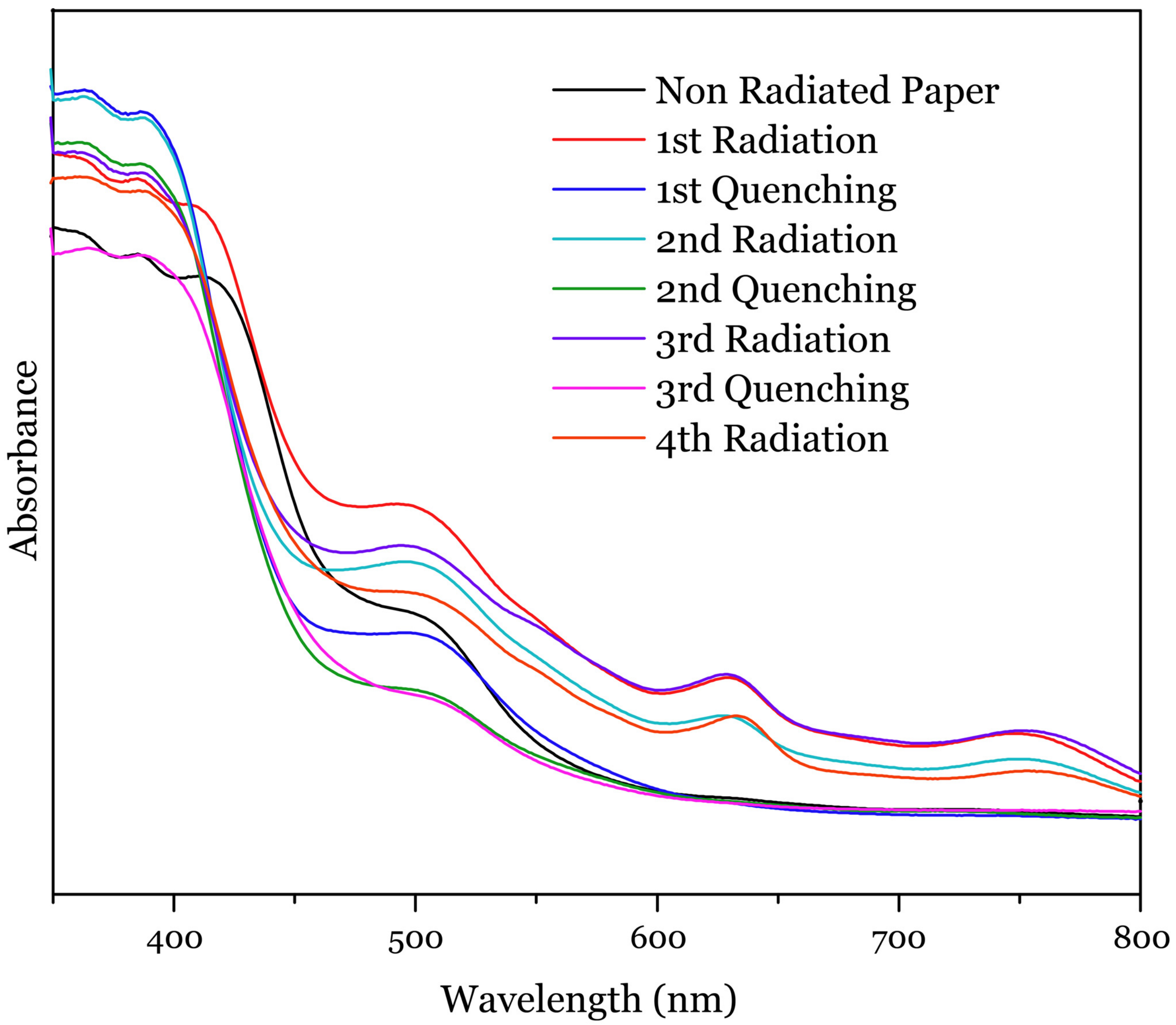

Figure S15: UV-vis study of Mg-NDI coated paper over consecutive cycles, showing good reversibility in each cycles.

S8: Electron Paramagnetic Resonance (EPR) studies

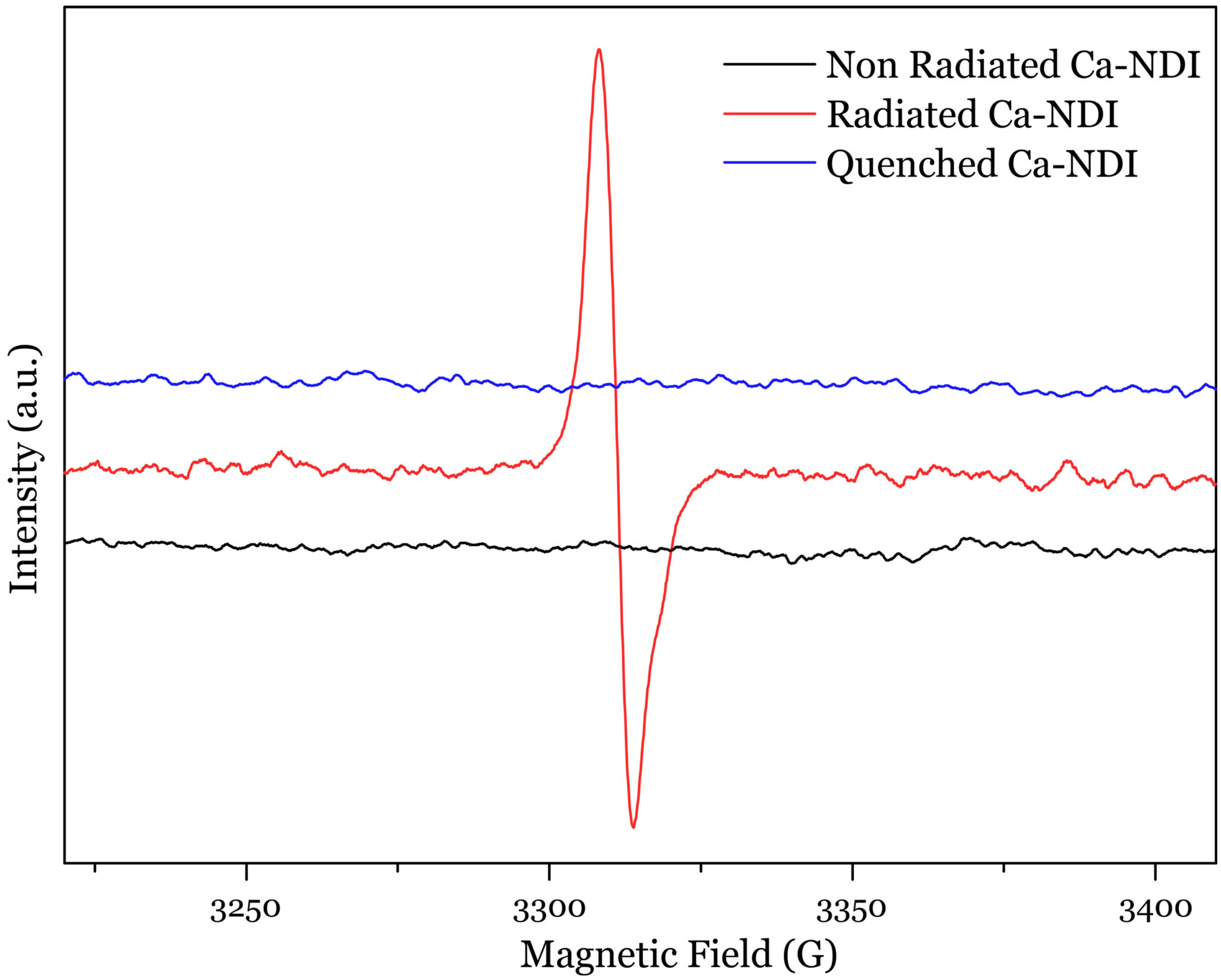

Figure S16: EPR spectra for Ca-NDI MOF showing generation of radical species because of sun-light irradiation and subsequent quenching in dark.

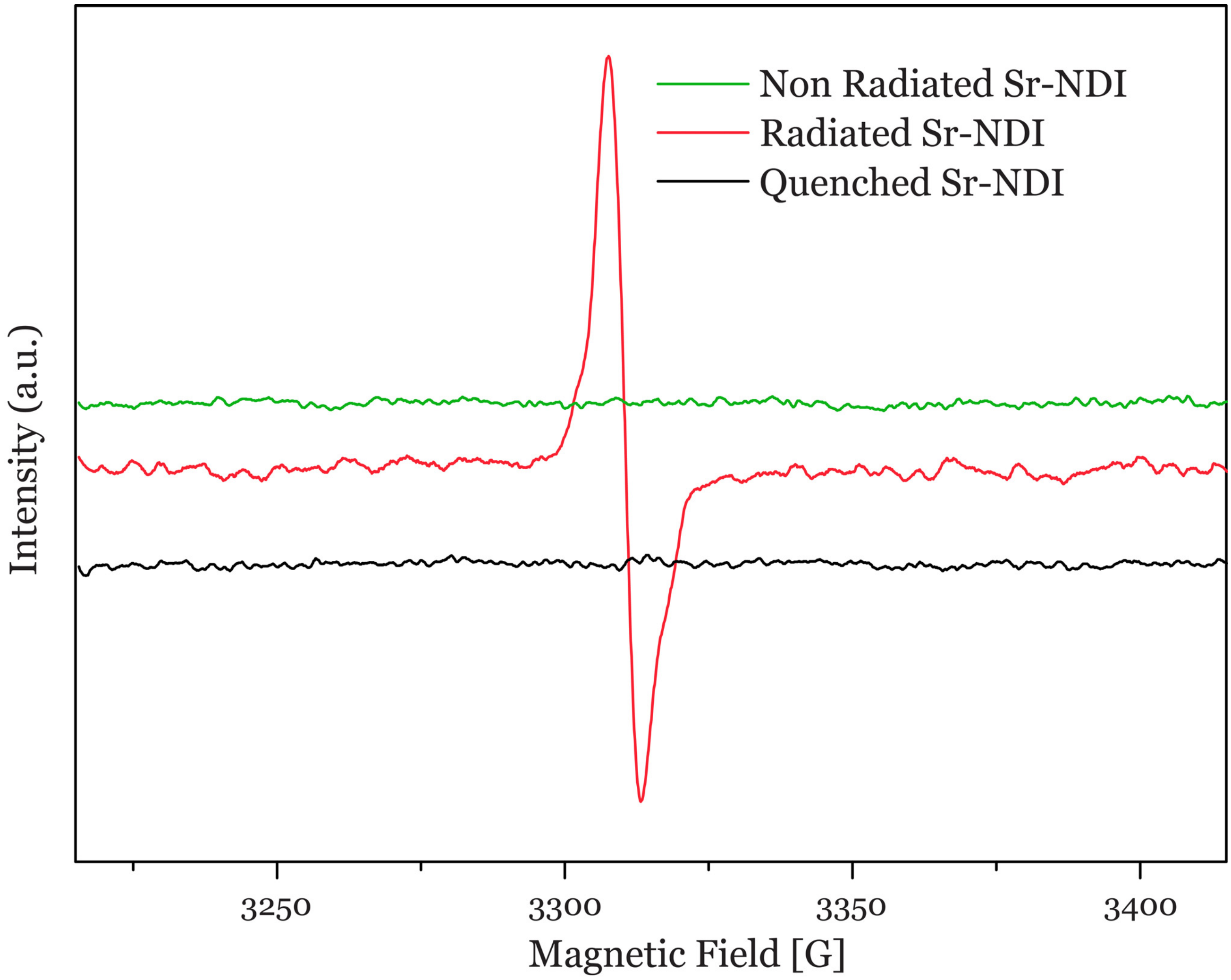

Figure S17: EPR spectra for Sr-NDI MOF showing generation of radical species because of sunlight irradiation and subsequent quenching in dark.

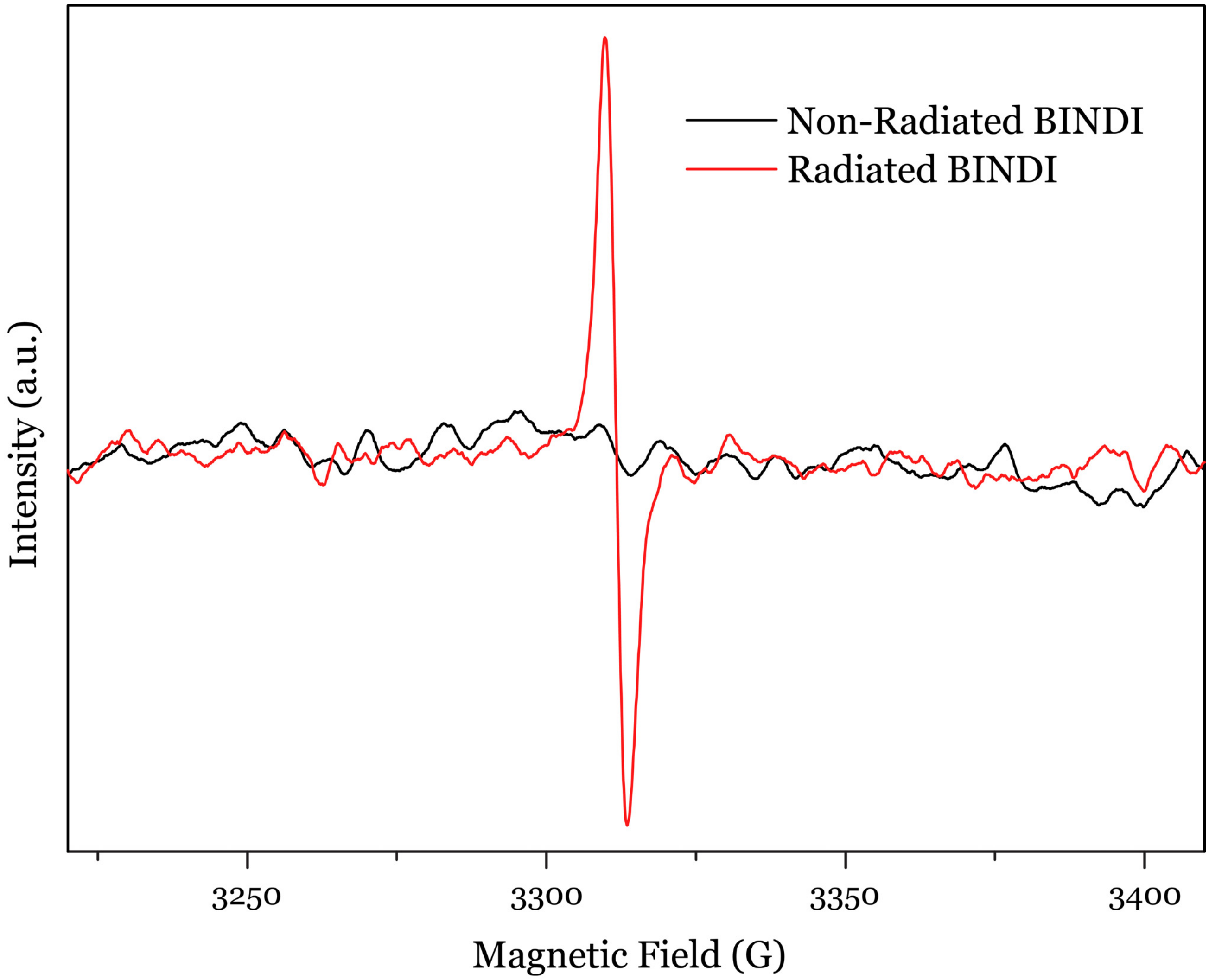

Figure S18: EPR spectra for BINDI Linker showing generation of radical species because of sun-light irradiation.

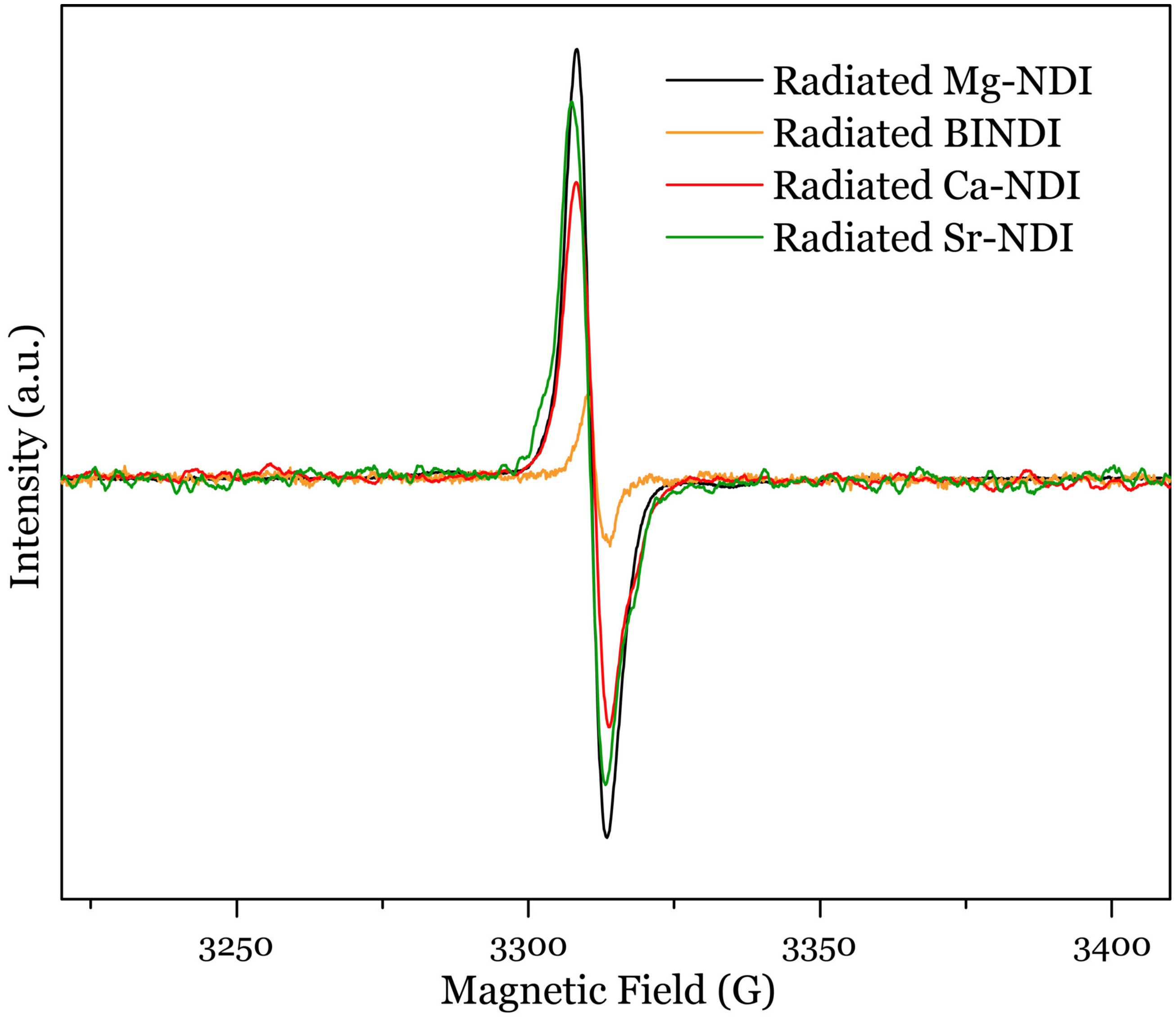

Figure S19: Comparison of all the EPR spectra for materials obtained after sunlight irradiation showing the same nature of the generated radical species.

S9: Photochromic property of BINDI

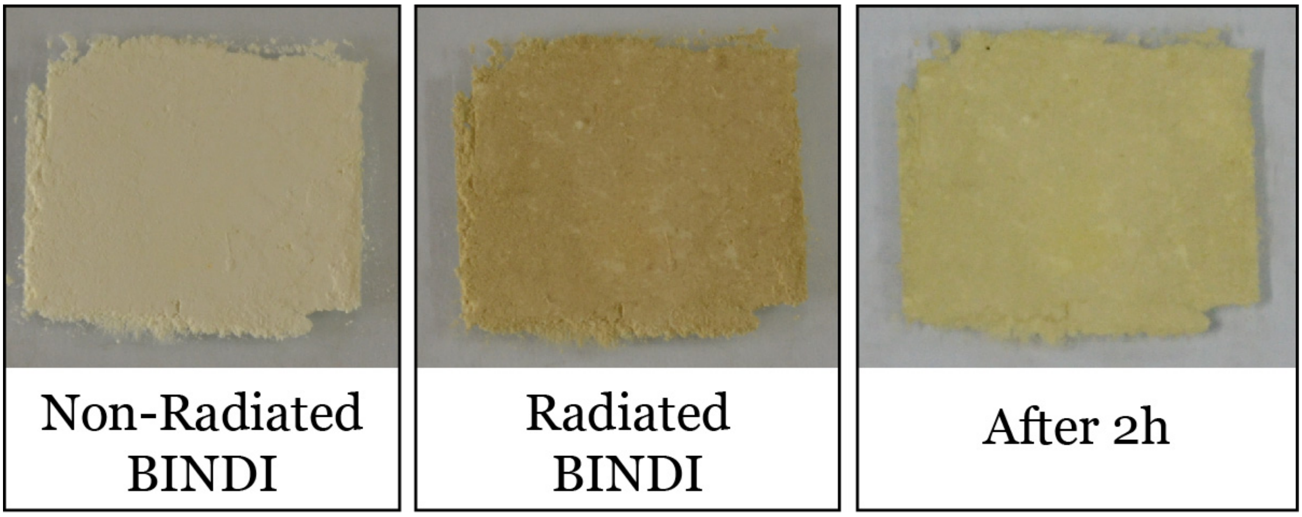

Figure S20: Colour change of free BINDI ligand under sunlight irradiation showing low contrast. The colour mostly reverts back to the original colour within a short period of time.

S10: SEM micrograph of Mg-NDI coated paper

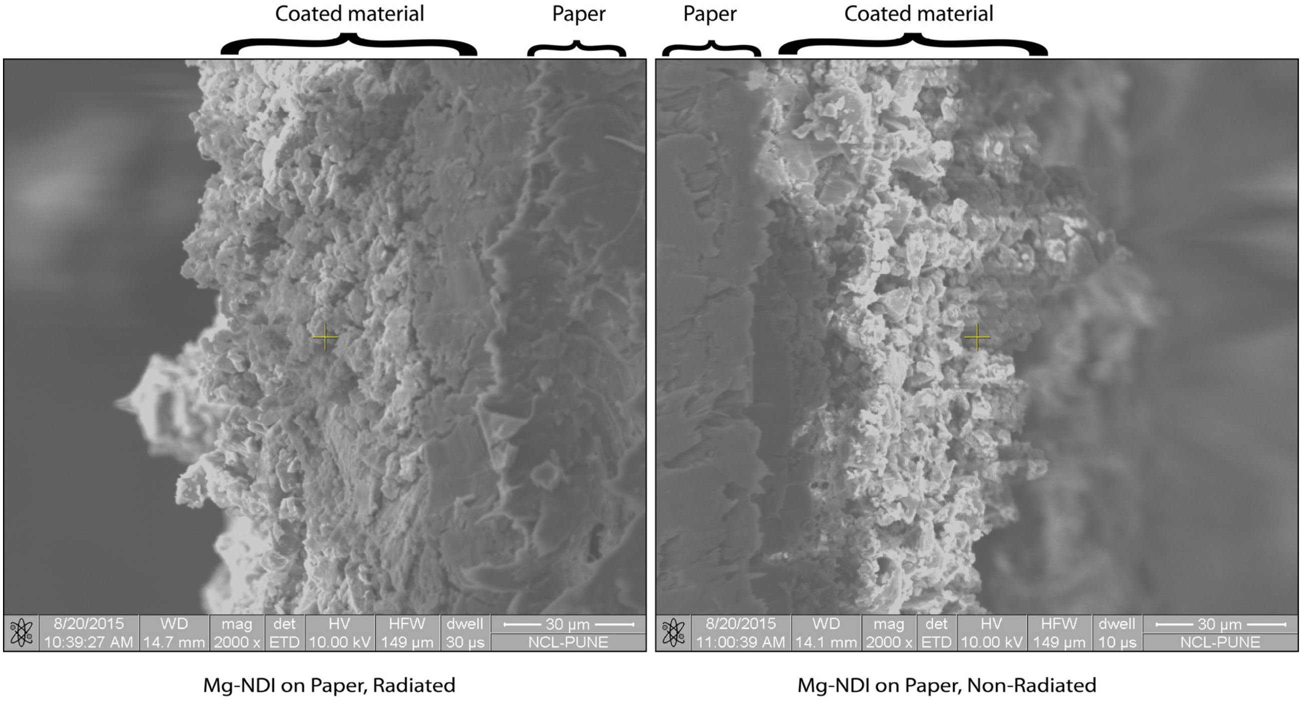

Figure S21: SEM images of the Mg-NDI coated paper before and after sunlight irradiation. The crystalline morphology of the coated materials remains after sunlight irradiation which is similar to pristine Mg-NDI crystals.

S11: Resolution test of printing with Mg-NDI coated paper

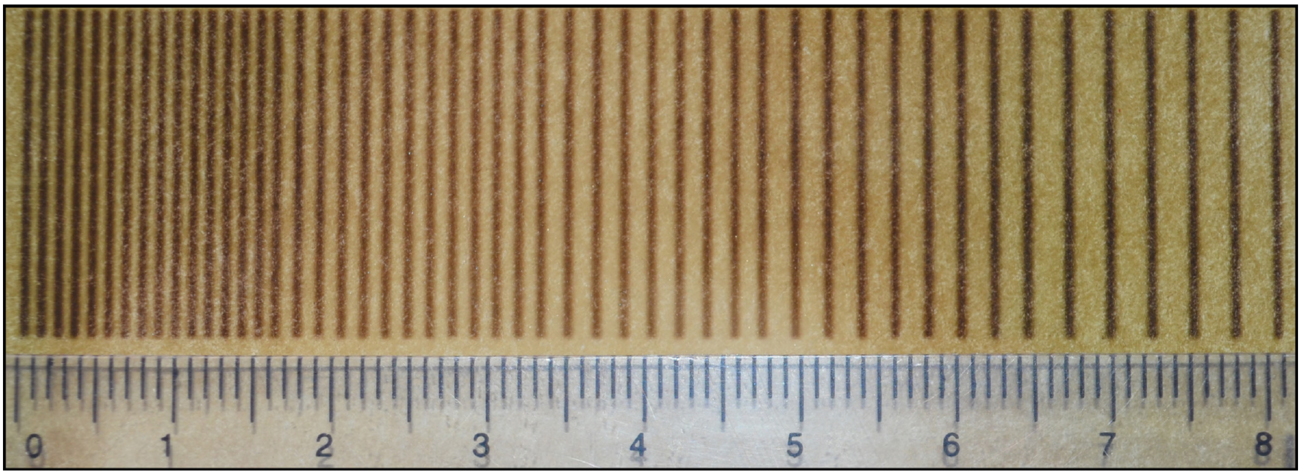

Figure S22: Resolution test of the printing on Mg-NDI coated paper with closely spaced parallel lines.

S12: Detection of 1D Barcode

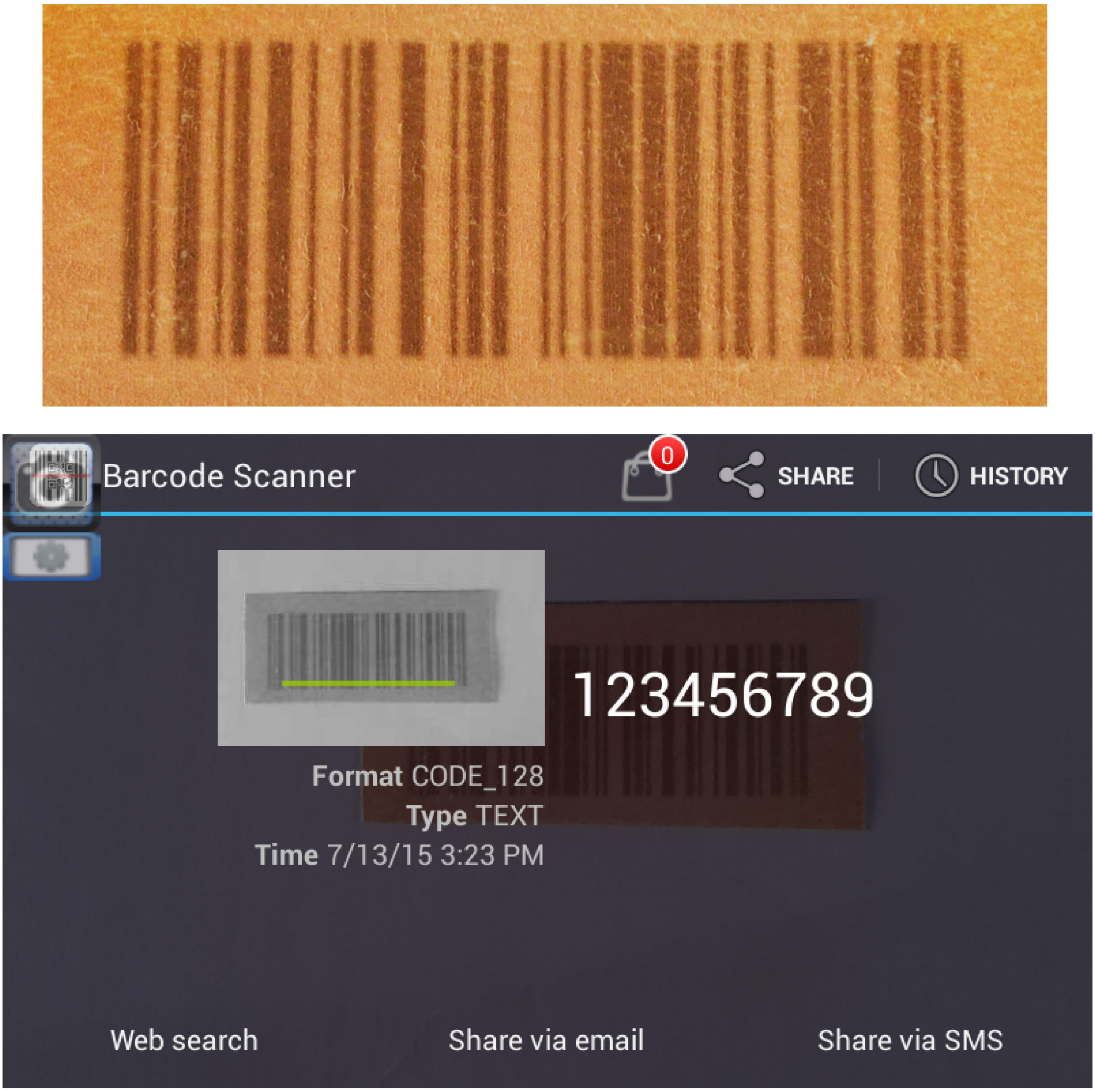

Figure S23: 1D barcode printed on Mg-NDI coated paper and the screenshot taken from a smartphone showing the decoding of the embedded code.

S13: Stability of the Mg-NDI coated paper towards mechanical deformation

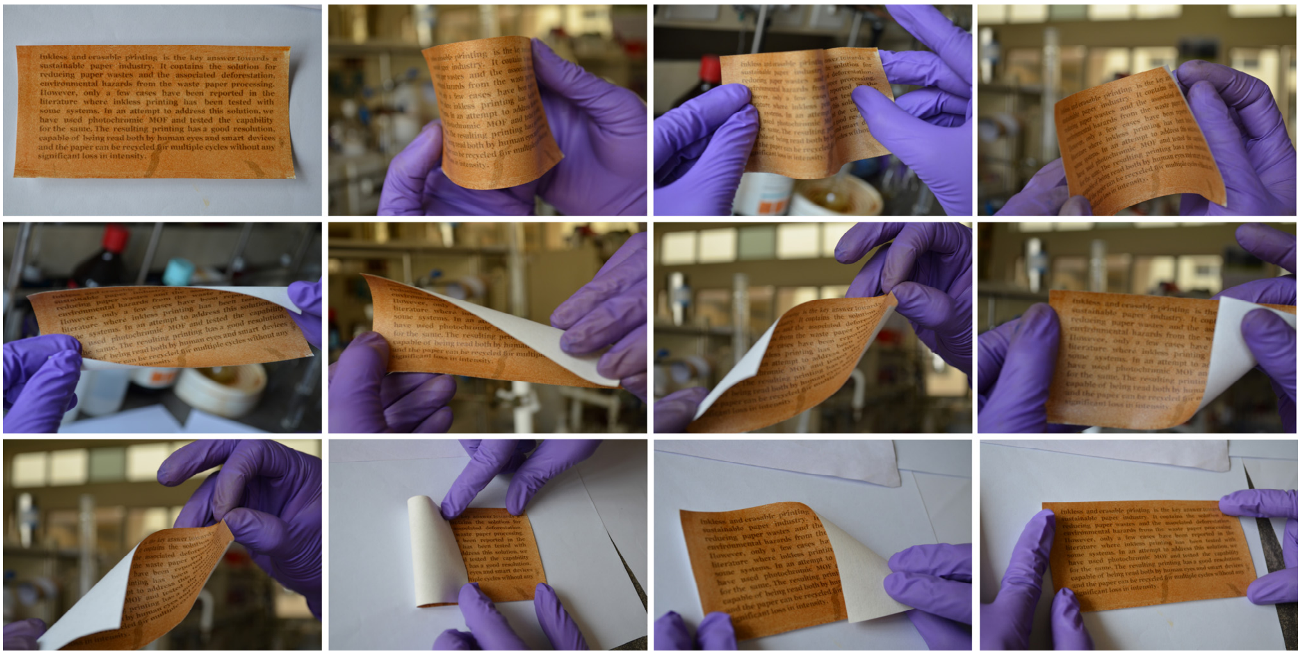

Figure S24: Mechanical Deformation of a Mg-NDI coated paper after printing content on it. It was observed that the contents remained clearly visible even after all kind of deformations applied to it.

S14: Preparation of Stencils

Stencils have been used to control the incidence of light on the MOF coated papers. Thus, any object capable of allowing passage of light through the required path can be used as a stencil for printing. Such objects include paper cut as per desired outline, suitable photomask, etc. However, for acheiveing a precise impression of the desired content, we have used the filtering of light through a inversely printed object on a transparent sheet. Thus, by printing the inverted object on a polyurethane sheet, we have generated a precise impression with sharp lines, edges that are legible not only to human eyes, but also to smart devices in the form of version-5 QR code. However, for large scale application, we are in process of developing new devices that can spontaneously and continuously control the incidence of light on the photochromic media, without requirement of any stencil.

S15: References

1. (a) Cairns, A. J.; Perman, J. A.; Wojtas, L.; Kravtsov, V. C.; Alkordi, M. H.; Eddaoudi, M.; Zaworotko, M. J. *J. Am. Chem. Soc.* **2008**,*130*, 1560. (b) Nelson, A. P.; Farha, O.K.; Mulfort, K. L.; Hupp, J. T. *J. Am. Chem. Soc.* **2009**, *131*, 458. (c) Perman, J. A.; Cairns, A. J.; Wojtas, L.; Eddaoudi M.; Zaworotko, M. J. *CrystEngComm* **2011**, *13*, 3130. (d) Han, L.; Qin, L.; Xu, L.; Zhou, Y.; Sun, J.; Zou, X. *Chem. Commun.* **2013**, *49*, 406.
2. CrysAlisPro, Version 1.171.33.66; Oxford Diffraction Ltd.: Abingdon, U.K., **2010**.
3. G. M. Sheldrick, (**1997**). *SHELXS '97* and *SHELXL '97*. University of Göttingen, Germany.
4. A. L. Spek (**2005**) PLATON, *A Multipurpose Crystallographic Tool*, Utrecht University, Utrecht, The Netherlands.
